# Alexithymia as a mediator between adverse childhood events and the development of psychopathology: a meta-analysis

**DOI:** 10.3389/fpsyt.2024.1412229

**Published:** 2024-07-01

**Authors:** Lorenz Kick, Daniel Schleicher, Angelika Ecker, Stephanie Kandsperger, Romuald Brunner, Irina Jarvers

**Affiliations:** ^1^ Department of Psychiatry and Psychotherapy, University of Regensburg, Regensburg, Germany; ^2^ Department of Child and Adolescent Psychiatry and Psychotherapy, University of Regensburg, Regensburg, Germany

**Keywords:** child abuse, alexithymia, psychopathology, TAS-20, CTQ, SCL-90, BSI

## Abstract

**Introduction:**

Victims of child abuse have an elevated risk of developing mental health issues later in life. Several variables have been suggested as mediators of this correlation, but little is known about the possible influence of alexithymia. Alexithymia is a sub-clinical personality trait that manifests as difficulties recognizing and verbalizing emotions.

**Methods:**

In this study, two separate meta-analyses were conducted using questionnaire data, and Pearson correlations for overall effects were estimated.

**Results:**

The correlation between child abuse and alexithymia showed to be significant (*r* = .26), as did the correlation between alexithymia and general psychopathology (*r* = .44). Further analyses revealed no indication for possible publication bias. When investigating differences between various subtypes of child maltreatment, each subtype significantly correlated with alexithymia. Emotional abuse, emotional neglect, and physical neglect had stronger correlations than physical and sexual abuse.

**Discussion:**

These results suggest that alexithymia plays a mediating role, at least in part, in the relationship between experiences of child abuse and general psychopathology in adulthood. Therefore, alexithymia may be relevant to further research and deserves attention in the prevention of and therapy for mental health issues in victims of child abuse.

## 1 Introduction

### 1.1 Alexithymia and mental health

Mental disorders pose a growing challenge for individuals and society (1). Some aspects of personality can lead to psychological distress but are not considered mental disorders per se. Alexithymia is thought to be one such subclinical risk factor trait, as empirical studies have demonstrated a correlation with many clinical mental and physical symptoms (2, 3). Factor analyses suggest difficulties identifying one’s own feelings (DIF), difficulties describing one‘s own feelings (DDF), and an externally orientated style of thinking (EOT) as the core features of alexithymia. In the process of regulating emotion, EOT can be seen as a deficit at the attention stage, and DIF and DDF as deficits at the appraisal stage (4). The causes of alexithymia can be manifold, and it can have serious implications in personal and social life (5). Regarding prevalence, Salminen et al. (6) found that 12.8% of a random sample of the general population in Finland had a questionnaire score above the suggested cut-off value for the existence of alexithymia. Prevalence differed between the sexes: 9.6% among female participants and 16.6% among male participants. Using the same cutoff-value in a German sample, Franz et al. (7) found a prevalence of 8.9% for female participants and 11.1% for male participants.

Alexithymia has been linked to a variety of mental disorders. Leweke et al. (2) reported a higher prevalence of alexithymia in patients with mental illness (21.4%) compared to the general population, particularly for depressive disorders (26.9%). Elevated alexithymia levels have also been found in patients with eating disorders (8). In a review, Teixeira (9) estimated the rate of alexithymic individuals among patients with substance use disorder to be between 50% and 70%. Correlations also exist between alexithymia and other forms of addiction, such as to mobile phones or internet (10, 11). Karukivi et al. (12) reported a significant association between high alexithymia scores and anxiety. A higher prevalence of alexithymia is found among those with anxiety orders than in the general population. The DIF and DDF facets of alexithymia seem to be linked to panic disorder, post-traumatic stress disorder (PTSD), generalized anxiety disorder, and social phobia. The EOT facet is present in obsessive-compulsive disorder (13). Most research thus far has aimed to connect alexithymia with clinical diagnoses of mental disorders, whereas the current literature lacks studies about the effects of alexithymia on subclinical psychopathology.

The question of causality in the connection between alexithymia and mental health issues cannot be addressed by cross-sectional studies. Using a longitudinal design, Karukivi et al. (14) found that the Toronto Alexithymia Scale (TAS)-20 total score does not predict anxiety, depression, or heavy drinking among young adults. The only significant correlation was between the DIF subscale and the emergence of anxiety. Similarly, among pregnant women, alexithymia was not found to be a risk factor for depressive episodes, but emerged as a state during the episode (15). Honkalampi et al. (16) also found that baseline alexithymia does not predict the development of depressive episodes, but it does correlate with the severity of depression. In addition, alexithymia scores decreased as the depressive episode vanished. Weissman et al. (17) found that low emotional awareness, as measured by DIF and DDF, significantly correlated with general psychopathology in children and adolescents. Low emotional awareness also predicted increased psychopathology over time. Thus, specific correlations seem to exist between facets of alexithymia and mental disorders.

Following these observations, researchers differentiated subtypes of alexithymia based on the cause and time of onset (18). Primary alexithymia refers to alexithymia as a stable personality trait that a person developed in their early years, influenced by biological and environmental context factors. In contrast, secondary alexithymia can arise as a reaction to psychologically challenging life events. Primary alexithymia is very stable, characterized by an early onset, and patients do not respond well to treatment attempts aiming to attenuate alexithymic features. Secondary alexithymia can arise early if triggered by a life event, but it can also first appear at an older age. Secondary alexithymia is not as stable as primary alexithymia and, therefore, can be more successfully influenced by therapy (19). The concept of secondary alexithymia can explain the high rates of alexithymia found in patients with medical conditions (20). In many cases, alexithymia can develop as a reaction during an episode of a mental illness, but there are also hints that alexithymia may be a risk factor for mental disorders. For example, the positive correlation between PTSD and alexithymia is well documented (21). Complex PTSD symptoms in adulthood are more likely to arise after traumatic experiences in childhood (22), and the experience of early traumatic situations has been hypothesized to be crucial in the development of alexithymia (23–25).

### 1.2 Child maltreatment and alexithymia

Childhood is a sensitive phase of life in which trauma can heavily impact further development, and a frequent cause of childhood trauma is experiencing episodes of abusive behavior (26). The DSM-5 differentiates between several types of child maltreatment: neglect, physical abuse, psychological abuse, and sexual abuse. While abusive actions are defined as non-accidental acts that potentially can harm a child, neglect means omission by adults that deprives the child of needs (27). The term sexual abuse refers to acts involving a child that are intended to provide sexual gratification to an adult (27). Combining several meta-analyses of self-reported data, Stoltenborgh et al. (28) quantified the prevalence rates of different subtypes of abuse to be between 6.3% and 22.6%.

Leeb et al. (29) summarized the increased risk of a variety of psychopathological symptoms after experiencing abuse in childhood. Experiencing abuse as a child can oftentimes lead to repetitive negative thoughts, as well as symptoms of PTSD in adulthood (30, 31). Adults who had experienced abuse or neglect also have a higher probability of suffering from depression, bipolar disorder, generalized anxiety disorder, addiction, suicidality, panic disorder, phobias, eating disorders, and psychotic symptoms (32–34). Some forms of child abuse also correlate with alexithymia in adulthood. Zdankiewicz-Ścigała and Ścigała (35) reported significant correlations between alexithymia and emotional neglect, but not physical, emotional, or sexual abuse. Similarly, Aust et al. (36) reported a correlation between emotional neglect and alexithymia, but not other types of child maltreatment. Feyzioğlu et al. (37) found significant positive correlations between all subscales of the Childhood Trauma Questionnaire (CTQ) and the TAS-20, except for the subscale of sexual abuse. This is congruent with Paivio and McCulloch (38), who found that child abuse, with the exception of sexual abuse, correlates with alexithymia in college students. Other studies found a correlation between sexual abuse in childhood and alexithymia (39–41). There are several possible mechanisms that could cause the correlation between childhood maltreatment and adulthood alexithymia. For example, not dealing with one’s emotion after traumatic experiences could be a protective mechanism (42). Another possible explanation could be that child abuse and neglect is more common in dysfunctional families, where children do not have a chance to learn about emotion regulation (43). In a meta-analysis, Khan and Jaffee (44) reported *r* = .24 for emotional neglect, *r* = .23 for physical neglect, *r* = .21 for emotional abuse, *r* = .11 for physical abuse, and *r* = .14 for sexual abuse. However, the informative value of this meta-analysis may be limited as it showed high heterogeneity between the studies. It is likely that at least some of the inconsistency between the studies on child maltreatment and alexithymia stems from the heterogeneity of the measurement instruments used, as the different questionnaires may differ in reliability, validity, and emphasis on different components of the constructs. Overall, results are inconsistent regarding correlations between the different subtypes of child maltreatment and alexithymia, the impact of sexual abuse is especially unclear. A meta-analysis with satisfactory heterogeneity is needed.

### 1.3 Alexithymia as a mediator between child maltreatment and psychopathology

Investigating possible transdiagnostic mechanisms that link child maltreatment with psychopathology, Weissman et al. (45) found evidence that dysfunctional regulation of emotion may act as a mediator. The concepts of alexithymia and emotion dysregulation are closely related (4). In line with this assumption, Chung and Chen (46) found that, among healthy adolescents, child abuse correlated positively with difficulties in emotional processing, which again correlated positively with alexithymia. Several other studies have investigated alexithymia as a possible mediator between experiences of child abuse and several mental disorders, such as personality disorders, depression, or eating disorders (36, 47–49). The results were inconsistent for specific mental disorders, but evidence of a mediating effect of alexithymia on the correlation between experiences of child abuse and general psychopathology should be investigated further (17, 50, 51). Yet, it is important to keep in mind that with cross-sectional data, directions of effects cannot be differentiated. It is also particularly important to have consistency in the conceptualization of the investigated variables. Focusing on certain valid and proven questionnaires can help provide clarity about the concepts being studied and facilitate scientific discourse.

### 1.4 Aim of the study

A small number of studies have already investigated the influence of alexithymia on the relationship between child abuse and different types of psychopathologies. Yet, how strongly all of these variables are associated and if alexithymia can be seen as a mediator of the correlation between child abuse and general psychopathology is still unclear. In addition, it is not clear to what extent the subtypes of child maltreatment differ from each other in this context. In the past, studies reported contradictory results regarding the differentiation between subtypes of abuse and neglect. The role of sexual abuse is especially controversial. This heterogeneity might be attributed, at least to some degree, to the use of different measurement instruments. Therefore, a meta-analytic approach focusing on specific valid questionnaires can be useful by contributing to the current state of research, reducing heterogeneity, and estimating effect sizes over a large number of participants and different populations.

The present meta-analysis aimed to collect and analyze studies that provide valid and generalizable data on the correlation between experiences of abuse in childhood and the development of alexithymia, specifically to compare different types of child abuse regarding their gravity as risk factors. This analysis also examines the correlation between alexithymia and psychopathology. By including not only psychiatric, but also healthy samples, and by focusing on a general factor of distress and psychopathology instead of psychiatric diagnoses, findings should be transferable to the general population. Following an observational approach, no interventions were compared. This study may help explain contradictory findings and further understand the interactions between these aforementioned variables. Furthermore, the size of the effects can be quantified by analyzing a large sample, and conclusions can be drawn about how alexithymia can be seen as a risk factor for the development of psychopathologies in the general population.

This study aimed to investigate the hypotheses that experiences of child abuse are associated with higher alexithymia scores (r > 0), and that higher alexithymia scores are again associated with higher general psychopathology (r > 0) using meta-analytic methods. Furthermore, correlations between alexithymia and different types of child abuse were examined. These comparisons may help detect differences between the types of child abuse regarding their significance as risk factors for alexithymia. Although analyzing specific facets of alexithymia is important and has been highlighted in several studies for its relationship with psychopathology, we decided against facet-level analysis in the present meta-analysis. This decision was based on the low reliability of some TAS-20 subscales (52, 53), which could potentially confound individual analyses.

## 2 Methods

### 2.1 Questionnaires

Child maltreatment, alexithymia, and psychopathology are scientific terms that have been conceptualized in different ways over the years. To ensure that the factorial structure of the data will be comparable between studies and allow for assumptions about subtypes, an *a priori* restriction was made regarding the questionnaires used in this meta-analysis. For each of the three variables, one widely used questionnaire with good psychometric properties was selected. Regarding experiences of child abuse and neglect, only studies using the CTQ were included (54). The TAS created by Taylor et al. (55) is the most common questionnaire to assess alexithymia and, therefore, was selected to be mandatory for inclusion in this analysis. As an indicator of distress and general psychopathology, the Global Severity Index (GSI) of the Symptom Check List-90-Revised (SCL-90-R) or its short form, the Brief Symptom Inventory (BSI), was used. Though this approach allowed for comparability between studies that used the same measures, it may limit the generalizability of the findings to studies that used different measures. Potential limitations and biases associated with this approach are discussed in the limitations section.

### 2.2 Study selection process

Following the PRISMA guidelines developed by Liberati et al. (56), suitable articles regarding the relationship between child abuse or neglect and the development of alexithymia were searched for in the databases PSYNDEX, PubMed, PsycINFO, and Google Scholar between October 2022 and May 2024. The keywords used to identify studies were “child abuse/neglect/maltreatment” and “alexithymia”. Depending on the search functions of the databases, the wording could have differed. The exact keywords for each database can be found in the **Supplementary Material** (**Supplementary Table 2.2.1**). In addition, the number of results differed greatly. The search on Google Scholar yielded over 7,000 results, whereas the other three databases together had only 321 studies. The first 200 results from Google Scholar were included in the screening, as the studies further in ranking were outside the scope of the search and unrelated to the topic.

**Figure 1:**
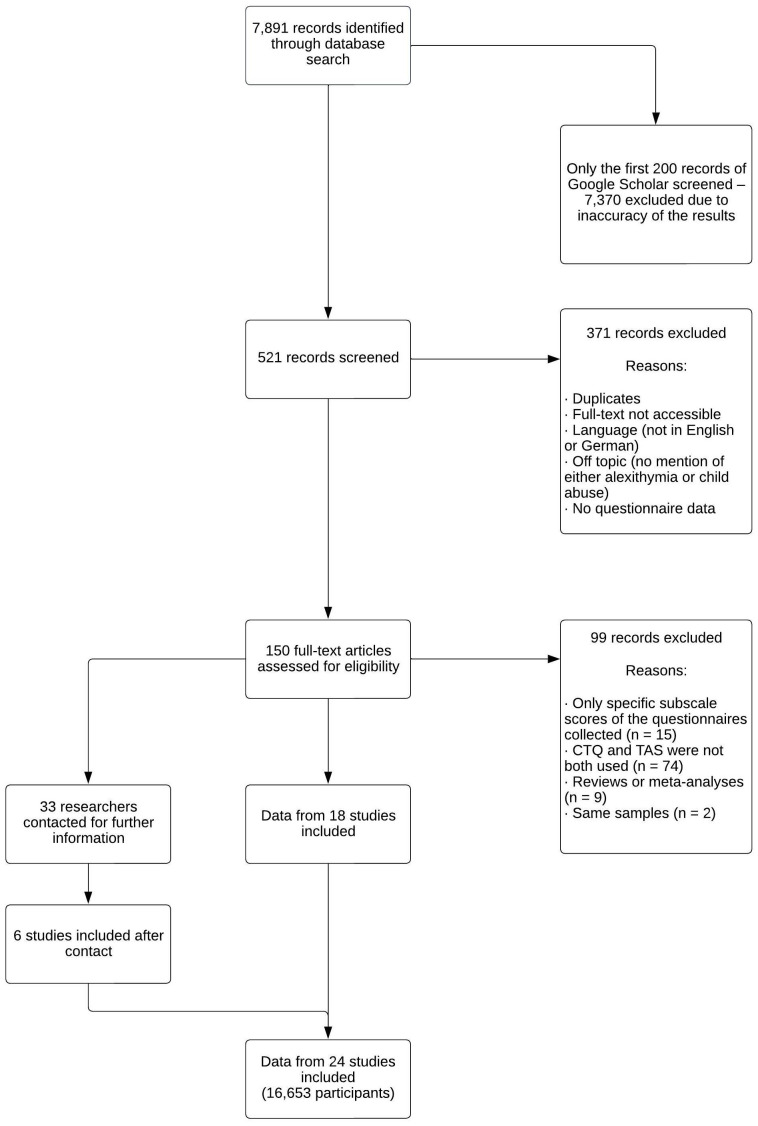
Study selection process for investigating the relationship between child maltreatment and alexithymia.

After identifying studies, they were screened for further information. Regarding data collected in the studies, alexithymia scores should be measured using the TAS, and child maltreatment should be queried with the CTQ. The correlation of the two questionnaires had to be quantified. Approximately 70% of the identified studies were excluded because they were duplicates, not accessible, or did not investigate both child abuse and alexithymia. Full-texts of the remaining studies were assessed, and approximately 70% of these studies were excluded. Reasons for exclusion were that completely different questionnaires or only some subscales of the CTQ and TAS were used, or that several articles were based on the same sample. In addition, reviews and meta-analyses were excluded. Finally, 18 studies were included in the meta-analysis. Several studies assessed CTQ and TAS but did not quantify the correlation in the article. Thirty-three researchers were contacted for further information, allowing six more studies to be included after responses from the author, for a total sample size of *n* = 16,653. The complete process of study selection is provided in **Figure 1**.

In a second step, the correlation between alexithymia and psychopathology was investigated using the same meta-analytic approach and browsing the same databases for suitable articles. Inclusion criteria were that general psychopathology should be measured with the SCL-90, SCL-90-R, or BSI, and alexithymia should be measured with a version of the TAS. In addition, correlation of the questionnaires had to be provided in the article. Therefore, keywords for the search were “alexithymia” and “SCL-90”, “SCL-90-R”, or “BSI”. The exact wording of the keywords used for each database can be found in the **Supplementary Material** (**Supplementary Table 2.2.2**). Again, only the first 200 articles found on Google Scholar were screened due to the inaccuracy of the results. One additional suitable article was found during literature research that was not found in any database. A total of 461 articles were screened. Approximately 60% of the studies were excluded after screening because they were not accessible, duplicates, not assessing both alexithymia and psychopathology, in a language other than German or English, or did not assess questionnaire data. After assessing the full-texts, approximately one-third were excluded because the questionnaires of interest were not used, they were reviews or meta-analyses, or they analyzed the same sample as another study that was already included.

**Figure 2:**
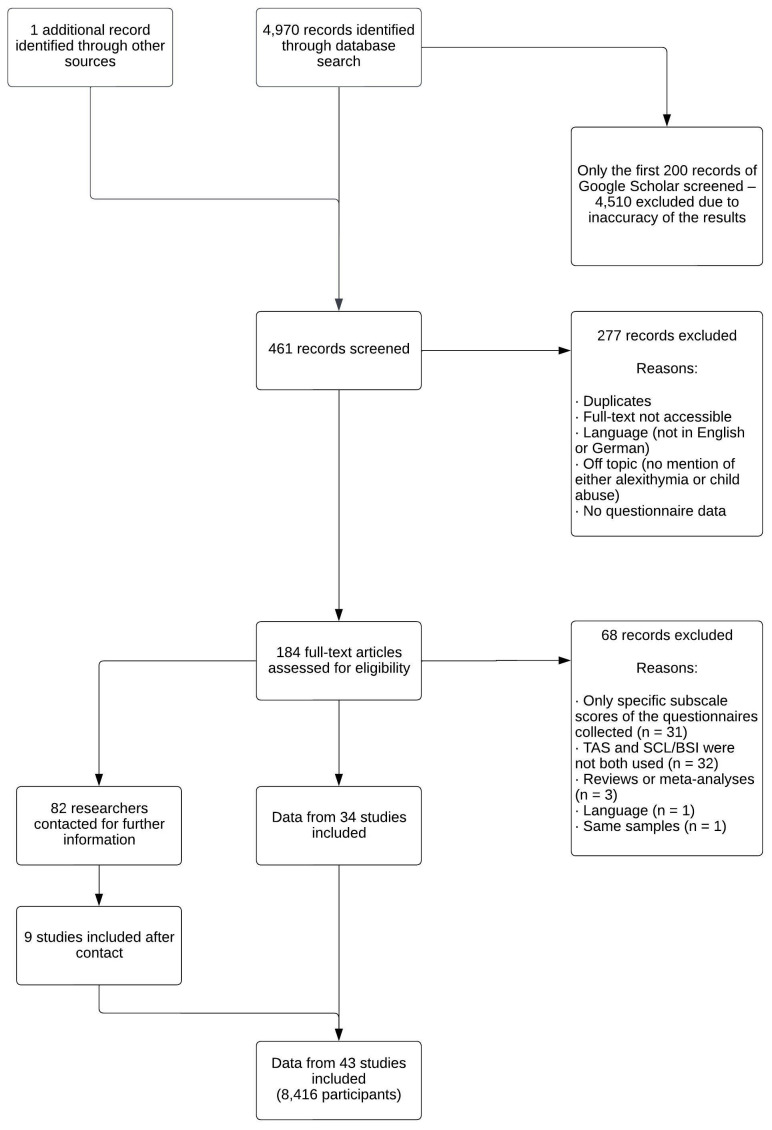
Study selection process for the correlation between alexithymia and psychopathology.

A total of 34 studies were cleared for inclusion right away, and 82 researchers were contacted because they assessed the questionnaires but did not report the correlation. Nine more studies could be included after responses were received from the authors for a total of 43 studies and a total sample size of *n* = 8,416 participants. When data were collected at several points over time in one study, data from the first assessment was used. Detailed information regarding the inclusion process is provided in **Figure 2**.

### 2.3 Data analysis

As Pearson’s correlation coefficient *r* is the most common indicator for quantifying correlations, it was also used in this study to estimate effect sizes (57). Most articles already reported the correlation as *r*, and the only other coefficient used in the collected articles was Kendalls’s *τ*, which was transformed into *r* (58). Correlations between the total scores from the CTQ/TAS and TAS/SCL were the main interest. Possible differences between various types of child abuse were accounted for by calculating and comparing correlations between CTQ subscale scores and the TAS overall score. Subsets of articles that reported these subscale correlations were created and analyzed separately. To weight effect sizes, the sample sizes of the studies were also coded. Other descriptive information that was coded was the mean age, percent female participants, country in which the study was conducted, type of sample, the exact versions of the questionnaires used, and mean scores on the questionnaires used.

The standard procedure for testing mediation as described by Baron and Kenny (59) is estimating regression coefficients for the influence of an independent variable on a mediator, the independent variable on a dependent variable, and the mediator on a dependent variable. If these influences are significant, mediation can be assumed. The effect size of the mediation can be estimated by controlling the mediator’s influence for the correlation between independent and dependent variables. If this correlation turns out to be nonexistent after controlling the mediator’s influence, the correlation between the independent and dependent variable can be completely attributed to the mediator.

In this study, child abuse is the independent variable, psychopathology the dependent variable, and alexithymia the mediator. As data for the three variables was taken from different samples, no full mediation analysis using regression models could be performed. The direct link between child abuse and psychopathology is not part of the analysis in this study. Nevertheless, mediation can be assumed if the estimates for the correlations between independent variable and mediator, and between mediator and dependent variable are significant (60). Instead of using regression models, the estimates for the correlation were computed by meta-analytic methods. A similar approach was described by van Dijk et al. (61), who also deducted mediational models from meta-analytic data. In the present study, a random-effects model was postulated to acknowledge differences between the studies and provide a more generalizable estimate of the overall correlation. I^2^ was calculated to analyze the heterogeneity of the studies; it describes the proportion of variation in the estimates that are caused by heterogeneity in the different study populations (62). To further investigate the moderating effect of important sample characteristics (health status of the populations, mean age, mean TAS score, percentage of female participants), subgroup analyses and meta-regressions were conducted. The possible influence of publication bias was estimated by analyzing funnel plots and computing a fail-safe N (63). All analyses were performed using R Statistical Software 4.2.2 (64). For meta-analytic calculations, the package “meta” was used (65).

## 3 Results

### 3.1 Child maltreatment and alexithymia

After completing the study selection process, 24 studies were included in the meta-analysis of the correlation between the experience of child abuse and alexithymia. **Table 1** gives an overview of information regarding the composition of the sample, questionnaires used, and Pearson correlations reported in the studies.

**Table 1:** Results of the meta-analysis for the correlation between child abuse and alexithymia.

Study	Nation	Sample	*n*	% female	CTQ edition	TAS edition	*r*
**Aust et al., 2013** (36)	Germany	Healthy	90	47	CTQ28	TAS-20	.26
**Carpenter & Chung, 2011** (66)	UK	OCD + control group	174	81	CTQ28	TAS-20	.42
**Chen et al., 2017** (67)	China	Prisoners	1,705	38	CTQ28	TAS-20	.21
**Chen et al., 2024** (68)	China	Amphetamine addicts	324	0	CTQ28	TAS-20	.06
**Feyzioğlu et al., 2022** (37)	Turkey	Healthy	435	69	CTQ28	TAS-20	.28
**Guhn et al., 2020** (69)	Germany	Persistent depression	34	59	CTQ28	TAS-20	.18
		Major depression	34	68			.40
		Healthy	34	59			.12
**Gülec et al., 2013** (70)	Turkey	Major depression + control group	50	62	CTQ28	TAS-20	.39
**Hoepfel et al., 2022** (71)	Germany	Healthy	58	58	CTQ28	TAS-20	.14
**Karaca Dinc et al.,** (50)	Turkey	Healthy	337	79	CTQ28	TAS-26	.33
**Kiefer et al., 2023** (72)	US	College students	294	100	CTQ28	TAS-20	.19
**Kopera et al., 2020** (73)	Poland	Alcohol abuse patients	255	10	CTQ28	TAS-20	.01
		Healthy	172	17			.37
**Li et al., 2024** (74)	China	College students	999	61	CTQ28	TAS-20	.48
**Liu et al., 2023** (75)	China	Healthy	134	69	CTQ28	TAS-20	.21
		Major depression	117	69	CTQ28	TAS-20	-.09
**Mlotek, 2019** (76)	Canada	College students	243	79	CTQ28	TAS-20	.41
**Paivio & McCulloch, 2004** (38)	Canada	Self-injuring college students + control group	41	100	CTQ28	TAS-20	.49
**Senkal & Isikli, 2015** (77)	Turkey	College students	417	76	CTQ28	TAS-20	.19
**Spitzer et al., 2009** (78)	Germany	Psychiatric patients	267	52	CTQ28	TAS-20	.18
**Strodl & Wylie, 2020** (79)	Australia	Healthy	332	88	CTQ28	TAS-20	.25
**Terock et al., 2016** (80)	Germany	Day-clinic outpatients	666	70	CTQ34	TAS-20	.19
**Terock et al., 2020** (81)	Germany	Healthy	5,574	52	CTQ34	TAS-20	.26
**Xie et al., 2021** (82)	China	College students	2,345	61	CTQ28	TAS-20	.36
**Zhang et al., 2020** (83)	China	College students	1,018	62	CTQ28	TAS-20	.31
**Zhang et al., 2021** (84)	China	Prisoners	362	0	CTQ28	TAS-20	.26
**Zou et al., 2016** (85)	China	Panic disorder	142	63	CTQ28	TAS-20	.37

Three studies reported correlations for different samples separately. For the analysis, these samples were treated as independent data entries. Therefore, 28 different samples were included. The minimum sample size was *n* = 34, and the maximum was *n* = 5,574. In total, a sample size of *n* = 16,653 was analyzed, including 9,157female participants (55%). The distribution of participants between continents based on the country of origin of the study was also analyzed (**Supplementary Material, Figure 2.1.1**). Most of the studies were conducted in either Asia or Europe, with no studies from South America or Africa.

**Figure 3:**
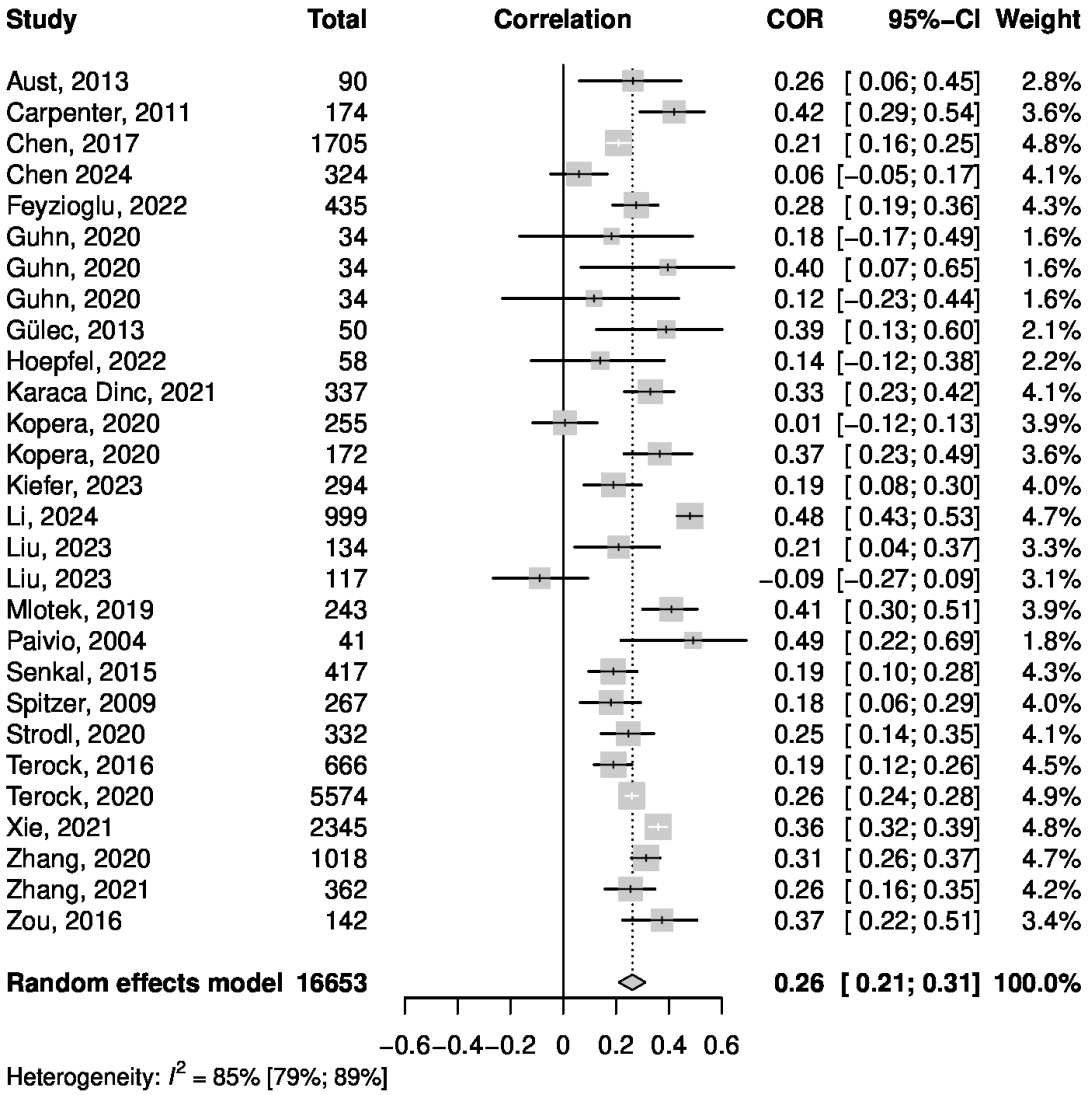
Forest plot summarizing the effects of the meta-analysis of the CTQ and TAS correlation.

The Pearson correlation between the CTQ and TAS reported in the studies was between *r* = -.09 and *r* = .49. **Figure 3** shows a forest plot that summarizes the meta-analytic random effects model. Inverse variance weighting was used to pool the studies. The overall correlation was estimated to be *r* = .26, with the 95% confidence interval (CI) of [.21;.31]. Regarding the heterogeneity of the studies, I^2^ = 84.7% indicates a high proportion of variance between the results of the meta-analysis relative to the total variance included in this analysis (86). Boxplot analysis revealed one significant outlier, which was the only study reporting a negative correlation. Omitting the outlier study with an influence analysis using the leave-one-out method led to a small change regarding the estimate for the correlation between CTQ and TAS (*r* = .27 compared to *r* = .26).

**Figure 4:**
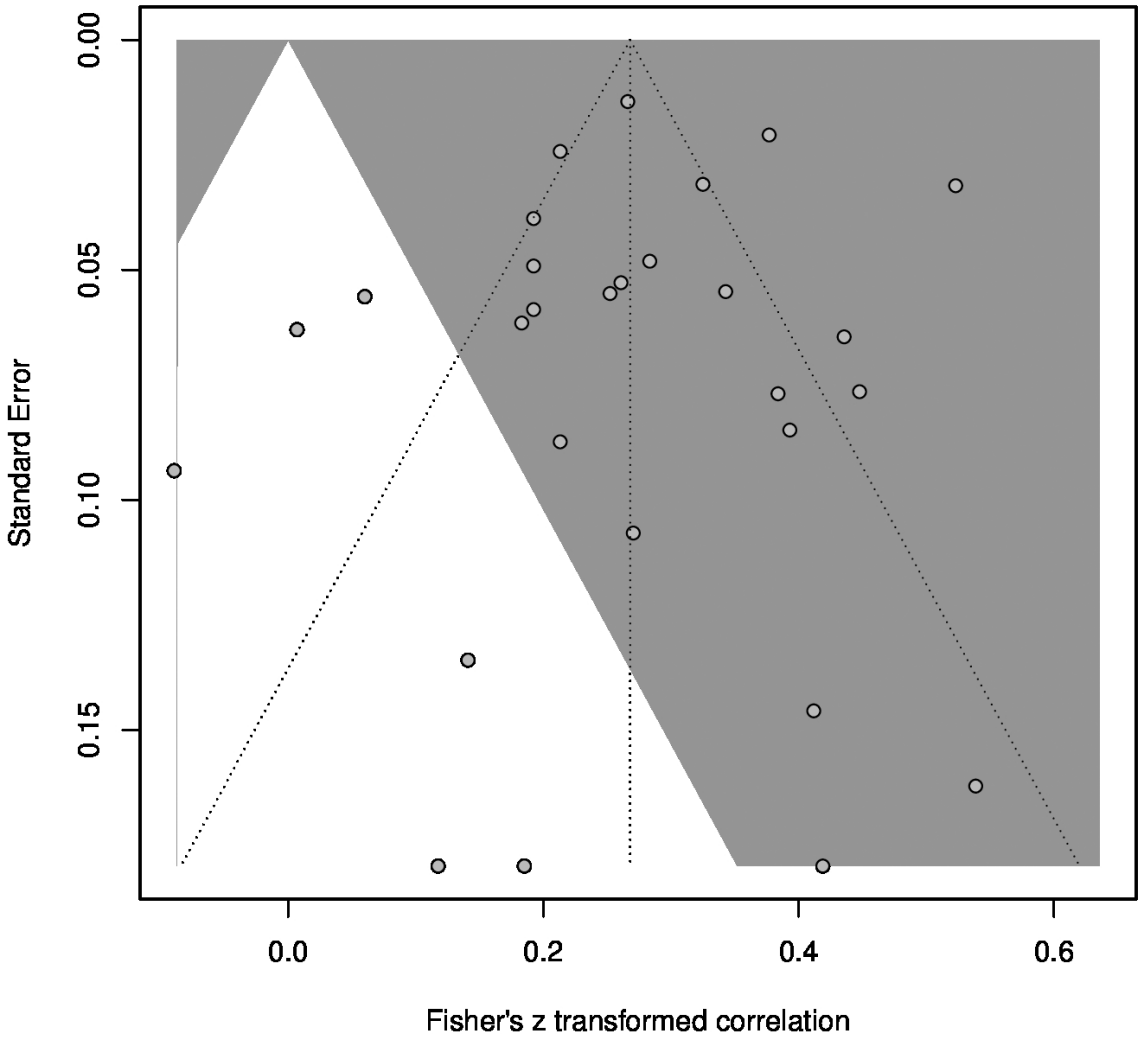
Funnel plot for the meta-analysis of the CTQ and TAS correlation. Studies in the grey area had significant results with α = .05.

A subgroup analysis revealed a significant difference between samples consisting of healthy and mentally ill subjects, with a higher correlation among healthy samples (*r* = .30) compared to samples with mental disorders (*r* = .15). Meta-regressions showed no significant influence on the correlation between CTQ and TAS for mean age, mean TAS score or percentage of female participants. Detailed information about the subgroup-analysis and meta-regressions can be found in the **Supplementary Material 3.1**. **Figure 4** shows a funnel plot used to detect possible influences of publication bias. The funnel plot shows no severe asymmetry for the included studies. This was confirmed by a linear regression test of funnel plot asymmetry, which showed no significant result (*p* = .56) (87). In addition, fail-safe N calculations suggested that 9,745 studies reporting no significant results would be required to bring the estimated overall *r* down to a barely significant level (88). Therefore, no effects of publication bias are suspected.

### 3.2 Subtypes of child maltreatment

Further analyses were conducted to compare differences between the subtypes of child maltreatment (physical abuse, emotional abuse, sexual abuse, emotional neglect, physical neglect) in regard to the impact on alexithymia scores. Separate meta-analyses were performed for each subtype. A total of 16 studies (17 different samples) that were used for the overall analysis also reported all five correlations between the CTQ subscales and TAS total score and were also included in the subset analyses. Six articles found during the literature search reported all of the subscale correlations but no overall correlation between the CTQ and TAS. In addition, two articles mentioned only specific correlations between subscales and the TAS and, therefore, could not be included in the overall analysis, but were included in the corresponding subset analysis. Thus, the results of 24 different articles contributed to the subset analyses. Forest plots and funnel plots for the five separate meta-analyses can be found in the **Supplementary Material** (**Supplementary Figures 2.1.3–2.1.12**). **Table 2** shows the number of analyzed articles, sample sizes, and estimated correlations between CTQ subscales and the TAS overall score for each of the five maltreatment subtypes. All five meta-analyses showed high heterogeneity with I^2^ > 81%. All five subtypes of child maltreatment significantly positively correlated with alexithymia scores.

**Table 2:** Comparison of CTQ subscales.

Maltreatment subtype	Number of studies	*n*	Pearson *r* [95% CI]	I^2^
**Emotional Abuse**	23	13,135	.23 [.16;.30]	82%
**Emotional Neglect**	24	14,450	.27 [.19;.35]	89%
**Sexual Abuse**	24	14,539	.13 [.07;.20]	83%
**Physical Abuse**	23	13,135	.16 [.08;.25]	86%
**Physical Neglect**	24	14,450	.24 [.17;.31]	81%

### 3.3 Alexithymia and psychopathology

Next, 43 studies that reported correlations between alexithymia and psychopathology were investigated using the meta-analytic approach. **Table 3** provides an overview of the information regarding the composition of the sample, questionnaires used, and Pearson correlation reported in the studies.

**Table 3:** Results of the meta-analysis of the TAS and SCL correlation.

Study	Nation	Sample	*n*	*%* female	TAS edition	SCL edition	*r*
**Barbosa et al., 2011** (89)	Portugal	Chronic urticaria + control group	86	83	TAS-20	SCL-90-R	.66
**Bilge et al., 2018** (90)	Turkey	College students	319	64	TAS-20	BSI	.60
**Bilotta et al., 2016 (study 1)** (91)	Italy	College students	100	49	TAS-20	SCL-90-R	.43
**Conrad et al., 2007** (92)	Germany	Chorioretinopathy	31	19	TAS-20	SCL-90-R	.11
		Control group	31	19			.44
**De Panfilis et al., 2015** (93)	Italy	Axis 1 diagnosis	167	61	TAS-20	SCL-90-R	.46
**Karaca Dinç et al., 2021** (50)	Turkey	Healthy	337	79	TAS-26	BSI	.57
**Evren et al., 2008** (94)	Turkey	Alcohol addiction	176	0	TAS-20	SCL-90-R	.6
**Flasbeck et al., 2017** (95)	Germany	Borderline personality disorder	37	100	TAS-20	SCL-90-R	-.07
		Control group	39	100			.59
**Imperatori et al., 2016** (96)	Italy	High TAS	36	83	TAS-20	SCL-90-R	.60
**Jones et al., 2004** (97)	US	Functional dyspepsia + control group	164	74	TAS-20	SCL-90-R	.44
**Kahramanol et al., 2018** (98)	Turkey	College students	434	56	TAS-20	BSI	.44
**Kerr et al., 2004** (99)	US	College students	56	75	TAS-20	SCL-90-R	.43
**Korkoliakou et al., 2017** (100)	Greece	Psoriasis	108	52	TAS-20	SCL-90-R	.27
**Köse et al., 2000** (101)	Turkey	Alopecia areata	18	0	TAS-26	BSI	.59
**Leweke et al., 2009** (102)	Germany	Mixed mental disorders	480	71	TAS-26	SCL-90-R	.4
**Li et al., 2020** (103)	China	College students	455		TAS-20	SCL-90	.45
**Li et al., 2022** (104)	Finland	Parents	659	79	TAS-20	SCL-90	.16
**Liang & West, 2011** (105)	US	College students	197	100	TAS-20	BSI	.41
**Linn et al., 2021** (106)	US	Alcohol	141	53	TAS-20	BSI	.56
**Ludwig et al., 2013** (107)	Suisse	Cancer	271		TAS-20	SCL-90-R	-.29
**Mannarini & Kleinbub, 2022** (108)	Italy	Anorexia	32	100	TAS-20	SCL-90-R	.57
		Healthy	32	56			.40
**Martinez-Sanchez et al., 2004** (109)	Spain	College students + teachers	332	77	TAS-20	SCL-90-R	.46
**Mikolajczak & Luminet, 2006** (110)	Belgium	College students	75	85	TAS-20	BSI	.31
**Pedrosa Gil et al., 2008** (111)	Germany	Somatoform disorder	32	72	TAS-26	SCL-90-R	.52
**Pedrosa Gil et al., 2008** (112)	Germany	Fibromyalgia	40	100	TAS-26	SCL-90-R	.76
**Porcelli et al., 2004** (113)	Italy	Mixed mental disorders	52	65	TAS-20	SCL-90-R	.40
**Renzi et al., 2020** (114)	Italy	Couples undergoing assisted reproductive techniques	79	100	TAS-20	SCL-90-R	.50
			39	0			.54
**Ritzl et al., 2018** (115)	Hungary	Personality disorder + control group	80	40	TAS-20	SCL-90-R	.77
**Saarijärvi et al., 2001** (116)	Finland	Major depressive disorder	120	55	TAS-20	BSI	.49
**Salcuni et al., 2021** (117)	Italy	Couples undergoing assisted reproductive techniques	118	100	TAS-20	SCL-90-R	.33
			118	0			.44
**Sayar et al., 2001** (118)	Turkey	Alopecia areata	31	0	TAS-26	BSI	.25
**Schäfer et al., 2002** (119)	Germany	Mixed mental disorders	419	65	TAS-20	SCL-90-R	.47
**Simonsen et al., 2021** (120)	Norway	Avoidant personality disorder	56	73	TAS-20	SCL-90-R	.43
**Simson et al., 2005** (121)	Germany	Mixed mental disorders	146	73	TAS-20	SCL-90-R	.37
**Simson et al., 2006** (122)	Germany	Mixed mental disorders	48	73	TAS-20	SCL-90-R	.57
**Subic-Wrana et al., 2002** (123)	Germany	Mixed mental disorders	240	72	TAS-20	SCL-90-R	.35
**Subic-Wrana et al., 2005** (124)	Germany	Mixed mental disorders	329		TAS-20	SCL-90-R	.37
**Tran et al., 2012** (125)	Austria	Psychosomatic inpatients	375		TAS-26	SCL-90-R	.58
**Tran et al., 2013** (126)	Austria	Healthy	670		TAS-20	BSI	.37
**Viganò et al., 2018** (127)	Italy	Chronic inflammatory bowel diseases	170	45	TAS-20	SCL-90-R	.27
**Vittori et al., 2022** (128)	Italy	Doctors	300	66	TAS-20	SCL-90-R	.47
**Wingbermühle et al., 2012** (129)	Netherlands	Noonan syndrome	39	59	TAS-20	SCL-90-R	.39
		Healthy	39	62			.43
**Zeeck et al., 2011** (130)	Germany	Binge eating and obesity + control group	63	100	TAS-20	SCL-27	.57

**Figure 5:**
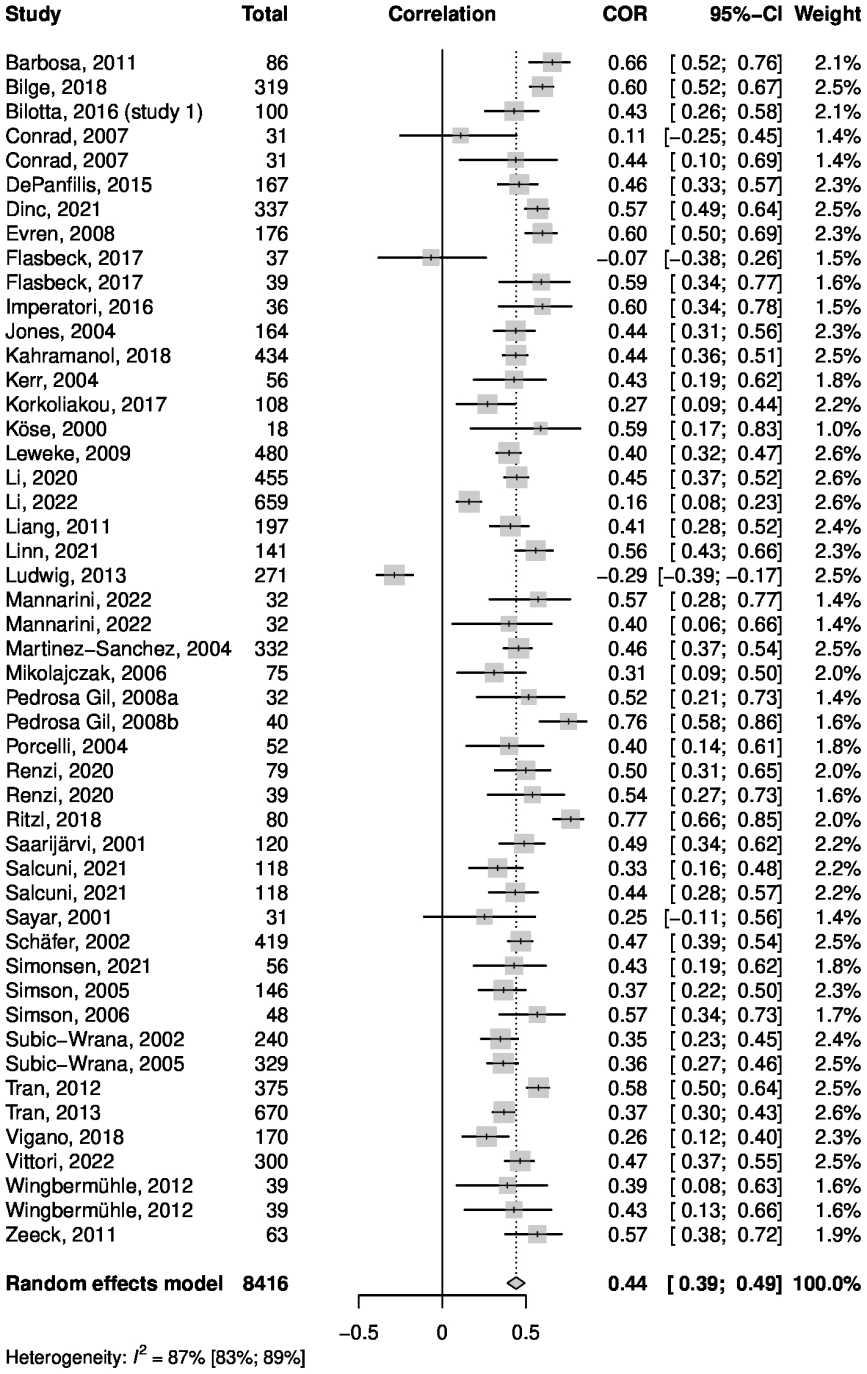
Forest plot summarizing the effects of the meta-analysis of the TAS and SCL correlation.

Six articles reported correlations for two separate samples, with a total of 49 data entries. Regarding sample sizes, the minimum was *n* = 18, and the maximum was *n* = 670. In total, a sample size of *n* = 8,416 was analyzed, including least 4,183 female participants (50%; sex not known for four samples). The distribution of participants between continents based on the country of origin of the study can be found in the **Supplementary Material** (**Supplementary Figure 2.1.2**). Only studies from Asia, Europe, and North America were identified for this analysis, with most participants living in European countries. For the Pearson correlation between the TAS and SCL/BSI, values between *r* = -.29 and *r* = .77 were reported. **Figure 5** shows a forest plot summarizing the meta-analytic random effects model. Inverse variance weighting was used for pooling. The overall correlation was estimated to be *r* = .44, with the 95% CI being [.39;.49]. I^2^ = 86.7% indicates high heterogeneity between the results of the samples. Boxplot analysis revealed two statistical outliers, which were the only two studies reporting a negative correlation. To examine the influence of these outliers, an influence analysis using the leave-one-out method was carried out. The results of the leave-one-out analysis with a focus on the two outlier studies are shown in **Table 4**.

**Table 4:** Results of the influence analysis for outliers.

Study omitted	*r* [95% CI]	I^2^
**Complete model (no omissions)**	.44 [.39;.49]	84.5%
**Flasbeck, 2017 (Borderline Group)**	.45 [.40;.50]	86.6%
**Ludwig, 2013**	.46 [.42;.50]	77.4%

These results show that excluding one of the two studies had little effect on the estimate of the overall *r* compared to the estimate for all studies together (*r* = .44). Yet, the sample from Ludwig et al. (59) seems to be a source of heterogeneity, as excluding the sample from the analysis would lead to a decrease in I^2^ from 86.7% to 77.4%.

**Figure 6:**
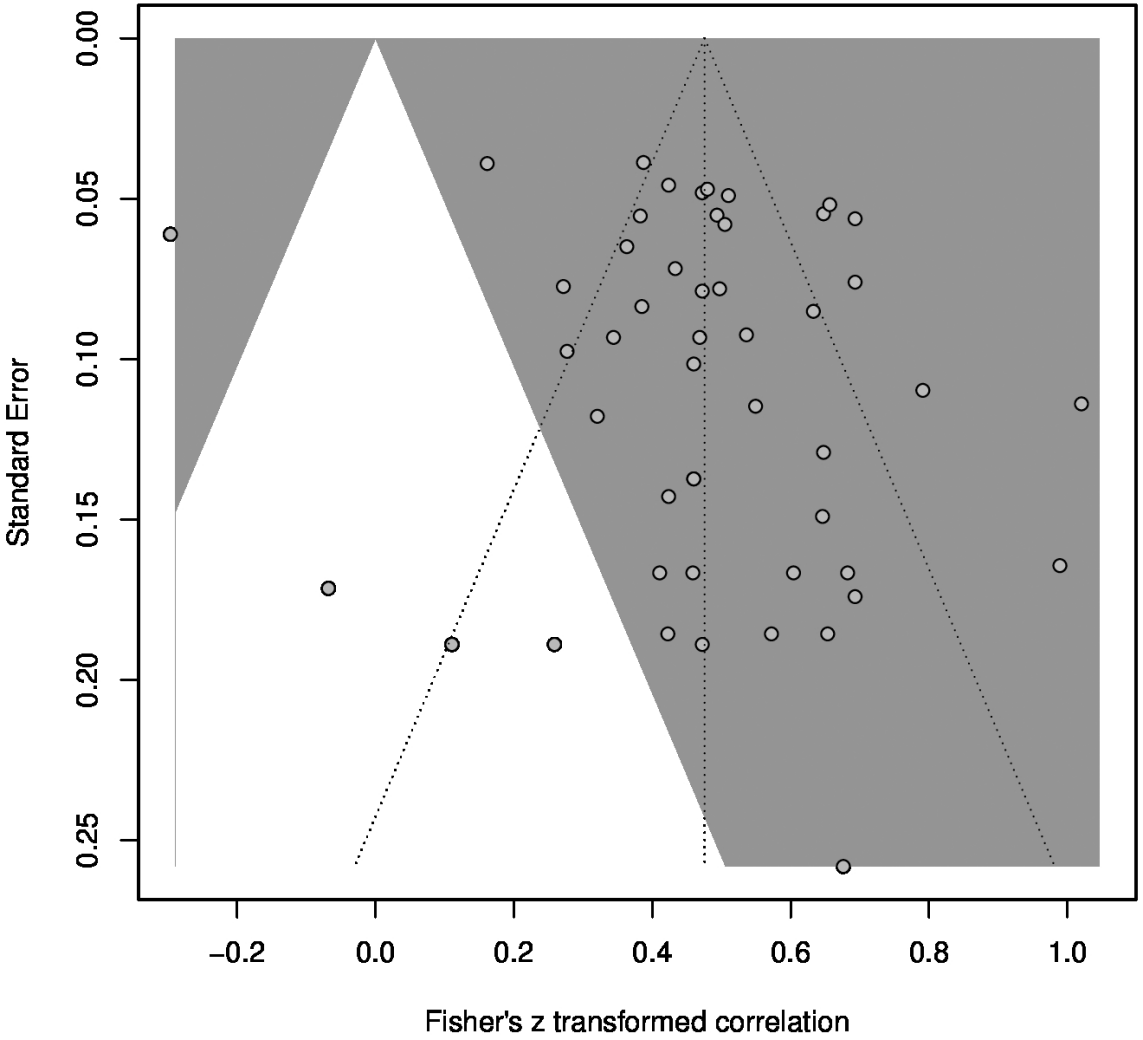
Funnel plot for the meta-analysis of the TAS and SCL correlation. Studies in the grey area had significant results with α = .05.

The funnel plot for the meta-analysis (**Figure 6**) showed no severe asymmetry of the included studies. This was confirmed by a linear regression test of funnel plot asymmetry, which showed no significant result (*p* = .21). In addition, fail-safe N calculations suggested that 40,112 studies reporting no significant results would be required to decrease the estimated overall *r* to a barely significant level. Therefore, no effects of publication bias are suspected. Furthermore, a subgroup analysis revealed no significant differences between samples consisting of healthy, physically ill, and mentally ill subjects. Meta-regressions showed no significant influence on the correlation between TAS and SCL for mean age, mean TAS score or percentage of female participants. Detailed information about the subgroup-analysis and meta-regressions can be found in the **Supplementary Material 3.2**.

**Figure 7:**
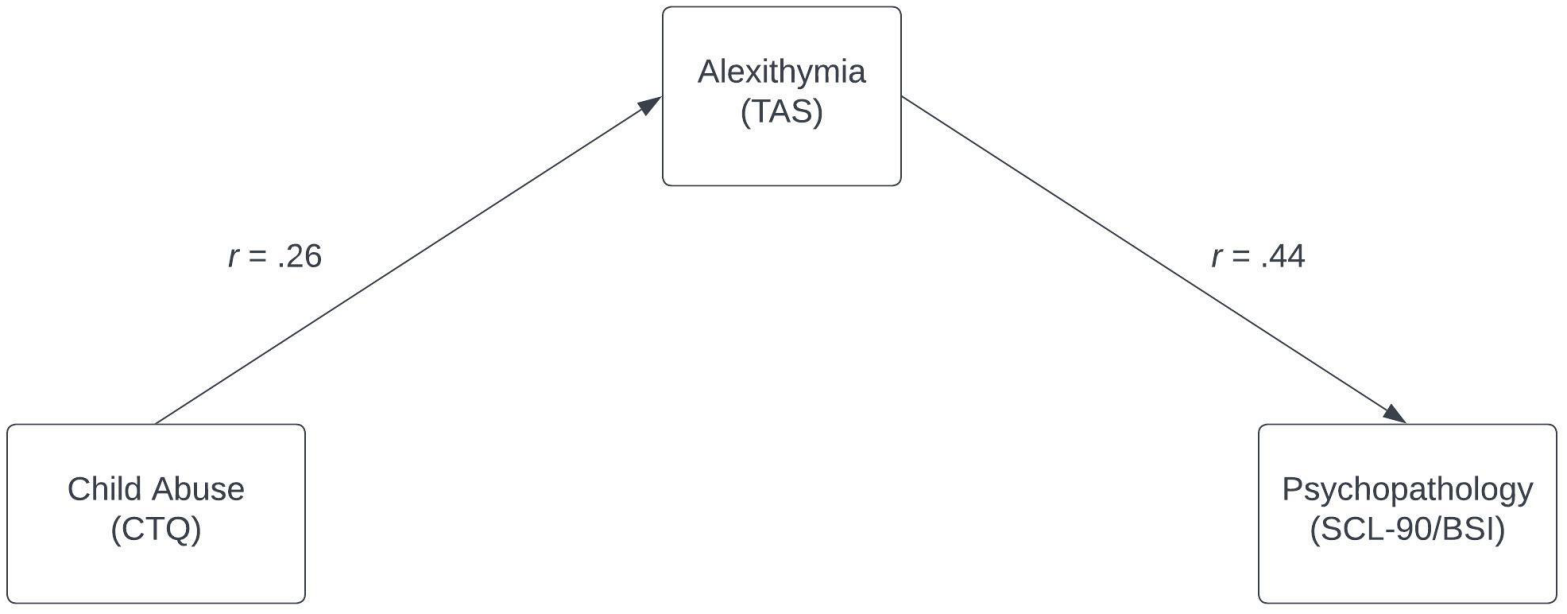
Model of mediation for child abuse, alexithymia, and psychopathology. The strength of the correlation is indicated by Pearson correlations as calculated in the meta-analyses. Both correlations are significant with α = .05.

Combining the results of the two separate meta-analyses, **Figure 7** summarizes the findings of this study in one proposed model of mediation.

## 4 Discussion

This study proposed alexithymia as a mediator of the correlation between experiences of child maltreatment and a general factor of psychopathology. Correlations for the indirect effect of the mediation model were estimated via meta-analyses. Results strongly suggest significant correlations between experiences of child abuse and alexithymia, as well as between alexithymia and general psychopathology. Combining the results of the two separate meta-analyses, alexithymia is suggested to at least partially mediate the correlation between child abuse and psychopathology (**Figure 7**).

This study only focused on the correlations between child abuse and alexithymia, and between alexithymia and psychopathology. Therefore, no statements can be made about the overall strength of the mediating effect, as the correlation between child abuse and psychopathology was not investigated. Furthermore, to conclusively test the model, it would be necessary to collect data about the three variables in one sample and analyze the pathways via regression models instead of using meta-analytic estimates. In particular, the timing of the onset of alexithymia and the onset of psychopathology cannot be deducted from cross-sectional data. Nevertheless, the meta-analysis provides a helpful summary of the current literature and could induce further experimental research by proposing a model of mediation.

Interestingly, the correlation between CTQ and TAS scores was significantly higher in healthy samples than in samples with mental illness. This could be due to a reduction in alexithymia in individuals who have undergone therapy for child maltreatment. Alternatively, it may suggest that the impact of childhood maltreatment on the development of alexithymia is more pronounced in the absence of mental illness. This is because, in the presence of mental disorders, various other factors could influence emotional processing. Supporting this notion, there were no significant differences in mean TAS scores between healthy samples and samples affected by mental illness. The findings of this study are in line with other research. Khan and Jaffee (39) also investigated the connection between child maltreatment and alexithymia, finding a Pearson correlation of *r* = .22. Correlations between the five subtypes of child maltreatment and alexithymia ranged from *r* = .11 (physical abuse) to *r* = .24 (emotional neglect). These values are slightly lower than the values found in this meta-analysis. Emotional neglect had the greatest influence in both studies. Nevertheless, both meta-analyses had a large overlap in the study pool, so similar results were expected. Though only the CTQ was investigated in this study, Khan and Jaffee included different questionnaires, leading to a higher number of observations but increased heterogeneity (*n* = 42,744, I^2^ = 92%). The findings from both studies support environmental factors in childhood playing a role in the development of alexithymia and indicate that health care professionals working with victims of child abuse should be aware of the possibility of elevated rates of alexithymia and the consequences that alexithymia may have for the treatment process. Eventually, alexithymia could also be targeted directly in therapy. Elevated rates of alexithymia are well documented in populations with mental disorders. Correlations with alexithymia scores have been found for depression, anxiety, addiction, and eating disorders (8, 9, 14, 131). More generally, Leweke et al. (2) reported a higher prevalence of alexithymia in a group of psychiatric patients compared to the general population.

In contrast to most of the research on the correlation between alexithymia and mental health problems, this study did not specifically focus on participants with certain mental disorders, but rather on a factor of general psychopathology. The idea of such a general factor is supported by the current literature (132). Though some of the samples included in this meta-analysis also came from a psychiatric background, approximately 53% of the sample consisted of healthy participants. Though there may still be an overrepresentation of participants with mental health issues, this study is one of the first to specifically estimate the correlation between alexithymia and (sub-)clinical psychopathology in the general population. This is further supported by the results of a subgroup analysis comparing healthy samples, samples with mental disorders, and samples with physical disorders. There were no significant differences in the correlation between TAS and SCL/BSI. If alexithymia and psychopathology potentially correlate even without the manifestation of a specific mental disorder, alexithymia may be a risk factor for mental illness rather than a consequence of it. In addition, the connection between alexithymia and psychopathology calls for more awareness of alexithymia in the treatment of mental disorders.

### 4.1 Differences between subtypes of child maltreatment

Though all subtypes of child maltreatment exhibited significant positive correlations with alexithymia, experiences of emotional abuse, emotional neglect, and physical neglect seemed to have a greater influence on the development of alexithymia than physical abuse or sexual abuse. As alexithymia is strongly associated with emotional intelligence, it seems plausible that emotional abuse or neglect can have a severe impact (133). Neglect, in particular, seems to lead to higher alexithymia. Neglect points to parents who fail to perceive or meet the emotional and physical needs of their children; thus, these children cannot learn the facets of emotional communication through role modeling (39). This is congruent with developmental theories that suggest that alexithymia can occur because certain emotional skills were not learned during childhood (134). Sexual abuse, compared to other subtypes of child maltreatment, had the smallest correlation with alexithymia. This finding is counterintuitive given the extensive literature about the severe and traumatic long-term consequences of sexual abuse (135). One possible explanation for this result could be that the term sexual abuse describes a spectrum of actions, which could have different implications for the development of alexithymia. Therefore, future studies could try to differentiate between the different types of sexually abusive actions (136). In addition, the duration and chronicity of maltreatment, age of the children, and possible development of PTSD should be taken into consideration as variables that can influence the effects of child maltreatment (137). Importantly, children often experience several subtypes of child maltreatment during their infancy (138). In these cases, it is complicated to differentiate between the subtypes and their separate influences on other variables.

### 4.2 Possible implications

Further validation of the proposed model, in which alexithymia mediates the correlation between experiences of child abuse and psychopathology, would lead to several inferences about the directions of action between the investigated variables. This model suggests that alexithymia is not innate in victims of child maltreatment, but develops due to experiences of maltreatment during childhood. Possible reasons may be that alexithymia serves as a coping strategy or that emotional skills cannot be learned due to a lack of appropriate role modeling or a lack of adequate reinforcement of emotional expression (39). Messina et al. categorized alexithymia that is shaped in early developmental phases of life as primary alexithymia rather than secondary alexithymia (19). In addition, they describe primary alexithymia as a risk factor for psychopathology. This is in line with the proposed model, as it assumes that problems with emotional processing - as indicated by higher alexithymia scores – are a predisposition for the occurrence of psychopathology, rather than alexithymia being developed secondarily as a reaction to mental health issues. Furthermore, assuming significant correlations between alexithymia and psychopathology not only for psychiatric, but also for healthy, populations supports the idea of alexithymia as a risk factor rather than a consequence of mental illness. Yet, prospective studies investigating alexithymia as a risk factor found little evidence that alexithymia can predict mental health problems and argue that these correlations remain to be investigated further (14, 139, 140).

The present findings also have practical implications. If alexithymia plays a mediating role in the connection between experiences of child abuse and psychopathology, the effects of alexithymia demand more attention in the treatment of psychiatric patients with a past history of child abuse. Prophylactic measures against alexithymia for children who are in treatment after experiencing abusive actions could also be an option to prevent the victims from developing mental disorders later in life. Though the literature on such prophylactic measures against alexithymia specifically is scarce, there are some suggestions for strategies to treat alexithymia in a therapeutic setting that could also be applied prophylactically. These approaches mainly center around learning the skills of recognizing and verbalizing emotions (141, 142). In addition, mindfulness-based interventions seem to have the potential to reduce alexithymia (143).

### 4.3 Limitations and outlook

This meta-analytic study produced estimates of the correlation between alexithymia and a subclinical factor of psychopathology that may be generalizable to the general population due to the high proportion of healthy samples in the analysis. In addition, strict preselection of questionnaires allowed for better inferences about underlying conceptual links between the variables by reducing heterogeneity, leading to the proposed model of mediation.

Nonetheless, several limitations must be considered when interpreting the results of this study. As data were restricted to specific questionnaires only, the generalizability of the study is reduced. Whenever child abuse, alexithymia, and psychopathology were discussed in this study, it was according to the conceptualizations proposed in these questionnaires. Though the chosen measures are the most commonly used questionnaires for alexithymia, child abuse, and psychopathology, potential shortcomings of these questionnaires also directly influence the results, such as the low reliability of the EOT subscale of the TAS (144).

For all meta-analyses in this study, heterogeneity (I^2^) was ≥ 75%, which is considered to be high. This suggests that the populations of the included studies were not homogenous due to, for example, regional or gender-based differences, and supports the assumption of a random-effects model (145). These differences between study populations seem plausible, as fluctuations in alexithymia scores have been found for different cultures and genders (146, 147). Regarding these cultural backgrounds, the greatest share of participants by far came from studies in European countries, followed by Asian countries. No suitable studies from Africa or South America were identified. This bias towards industrialized populations is often seen in psychological literature and constitutes a problem, as it impairs the generalizability of the results for populations from other regions (148). One possible starting point for improving future research could be to compare the effects for different populations, taking into consideration gender, ethnicity, or health status, among other factors.

Another weakness that could be targeted by future research is the cross-sectional nature of the data. Almost all of the data in this study came from articles that had only one measuring point, at which current values of alexithymia and general psychopathology, as well as retrospective data on experiences of child abuse, were queried. Thus, the assumptions of the proposed model that alexithymia and psychopathology develop after experiences of child abuse are questioned, as they could have already existed in infancy. For example, alexithymia may be an innate trait that impairs adaptive processing of experiences of child abuse, leading to the development of psychopathology. Another alternative hypothesis to explain the findings of this study could be that child abuse can lead to psychopathology, and alexithymia can develop as a reaction to already present mental health issues. To rule out these possibilities, a longitudinal approach that follows participants with several data collection points during the progression of childhood into adulthood would be necessary.

## 5 Conclusion

This meta-analytic study quantified the correlations between experiences of child abuse and alexithymia (*r* = .26) and between alexithymia and general psychopathology (*r* = .44). As both correlations were significant, alexithymia can be hypothesized to at least partially mediate the correlation between child abuse and psychopathology. This would indicate that the development of alexithymia could be seen as a risk factor for psychopathology in victims of child abuse, and that alexithymia should be targeted more heavily in therapeutic settings. Yet, the study design leaves room for improvement. To investigate the mediation model in as detailed a manner as possible, longitudinal data for all three pathways between child abuse and alexithymia, and both child abuse and psychopathological symptoms should be collected in the same sample. This would allow for more conclusive statements, and the effect of mediation could be quantified.

## Data availability statement

The original contributions presented in the study are included in the article/**Supplementary Material**. Further inquiries can be directed to the corresponding author.

## Author contributions

LK: Writing – original draft, Visualization, Validation, Methodology, Formal analysis, Data curation, Conceptualization. DS: Writing – review & editing, Validation, Methodology, Formal analysis, Data curation, Conceptualization. AE: Writing – review & editing, Validation, Conceptualization. SK: Writing – review & editing, Validation, Supervision, Project administration, Conceptualization. RB: Writing – review & editing, Validation, Supervision, Project administration, Conceptualization. IJ: Writing – review & editing, Validation, Supervision, Project administration, Methodology, Formal analysis, Data curation, Conceptualization.
