# Supplementary material for: Alexithymia as a mediator between adverse childhood events and the development of psychopathology: a meta-analysis
Source: Front Psychiatry. 2024 Jul 1;15:1412229. doi: 10.3389/fpsyt.2024.1412229 (PMC11246998; doi:10.3389/fpsyt.2024.1412229)
Supplement: Supplementary file 1 [file DataSheet_1.docx]

Supplementary Material

# Reference Lists for the Meta-Analyses

## Articles included in the meta-analysis between CTQ and TAS

Aust S, Härtwig EA, Heuser I, Bajbouj M. The role of early emotional neglect in alexithymia. Psychological trauma: theory, research, practice, and policy 2013; 5(3):225.

Brown S. Associations between child maltreatment types and past month substance use among emerging adults: The role of self-reported alexithymia and impulsivity. University of Kansas; 2019. Available from: URL: https://kuscholarworks.ku.edu/handle/1808/31369.

Carpenter L, Chung MC. Childhood trauma in obsessive compulsive disorder: the roles of alexithymia and attachment. Psychology and Psychotherapy: Theory, Research and Practice 2011; 84(4):367–88.

Chen L, Xu L, You W, Zhang X, Ling N. Prevalence and associated factors of alexithymia among adult prisoners in China: a cross-sectional study. BMC Psychiatry 2017; 17(1):287. Available from: URL: <https://link.springer.com/article/10.1186/s12888-017-1443-7>.

Chen J, Dong MW, Yang T, Rongrong Z, Yuqing L, Linanglun J, Fabing F, Shanshan T, Xiaotao W, Li W, Xiang YZ. Childhood abuse and craving in methamphetamine-dependent individuals: the mediating role of alexithymia. European Archives of Psychiatry and Clinical Neuroscience 2024; 1-9.

Feyzioğlu A, Taşlıoğlu Sayıner AC, Özçelik D, Tarımtay Altun F, Budak EN. The mediating role of early maladaptive schemas in the relationship between early childhood trauma and alexithymia. Curr Psychol 2022:1–11. Available from: URL: https://idp.springer.com/authorize/casa?redirect_uri=https://link.springer.com/article/10.1007/s12144-022-02988-1&casa_token=_f_4wbnqmzcaaaaa:fqa_h0xz2gsiiyqjv_djcgerrpnndijtqueg5_jven2dcog-qou3t4xa2pq3-fkz_f8wd0o8ehuabhtvvq.

Frewen PA, Dozois DJ, Neufeld RWJ, Lanius RA. Disturbances of emotional awareness and expression in posttraumatic stress disorder: Meta-mood, emotion regulation, mindfulness, and interference of emotional expressiveness. Psychological trauma: theory, research, practice, and policy 2012; (4(2)):152. Available from: URL: https://psycnet.apa.org/record/2011-06112-001.

Frewen PA, Pain C, Dozois DJA, Lanius RA. Alexithymia in PTSD: psychometric and FMRI studies. Annals of the New York Academy of Sciences 2006; 1071(1):397–400.

Guhn A, Merkel L, Hübner L, Dziobek I, Sterzer P, Köhler S. Understanding versus feeling the emotions of others: How persistent and recurrent depression affect empathy. Journal of Psychiatric Research 2020; 130:120–7. Available from: URL: https://www.sciencedirect.com/science/article/pii/s0022395620308554.

Güleç MY, Altintaş M, İnanç L, Bezgin CH, Koca EK, Güleç H. Effects of childhood trauma on somatization in major depressive disorder: The role of alexithymia. Journal of Affective Disorders 2013; 146(1):137–41. Available from: URL: https://www.sciencedirect.com/science/article/pii/s016503271200496x.

Hoepfel D, Günther V, Bujanow A, Kersting A, Bodenschatz CM, Suslow T. Experiences of maltreatment in childhood and attention to facial emotions in healthy young women. Scientific reports 2022; 12(1):4317.

Karaca Dinç P, Oktay S, Durak Batıgün A. Mediation role of alexithymia, sensory processing sensitivity and emotional-mental processes between childhood trauma and adult psychopathology: a self-report study. BMC Psychiatry 2021; 21(1):508. Available from: URL: <https://link.springer.com/article/10.1186/s12888-021-03532-4>.

Kiefer R, Orchowski LM, Raudales AM, Weiss NH. Role of Alexithymia in the Association Between Childhood Abuse and Risk Perception for Sexual Victimization in College Women. Journal of Interpersonal Violence 2024; 39(3-4):756-784.

Kopera M, Zaorska J, Trucco EM, Suszek H, Kobyliński P, Zucker RA et al. Childhood trauma, alexithymia, and mental states recognition among individuals with alcohol use disorder and healthy controls. Drug and Alcohol Dependence 2020; 217:108301. Available from: URL: <https://www.sciencedirect.com/science/article/pii/s037687162030466x>.

Li X, Wang X, Sun L, Hu Y, Xia L, Wang S, Guo C, Shi Y, Yuan X, Zhan K, Liu H. Associations between childhood maltreatment and suicidal ideation in depressed adolescents: The mediating effect of alexithymia and insomnia. Child Abuse & Neglect 2023; 135:105990.

Li M, Yuan Y, Cheng X, Wang Y, Xu Z. Childhood maltreatment and insomnia in college students: The role of alexithymia and psychological distress. Acta psychologica 2024; 243:104149.

Liu H, Wang W, Yang J, Guo F, Yin Z. The effects of alexithymia, experiential avoidance, and childhood sexual abuse on non-suicidal self-injury and suicidal ideation among Chinese college students with a history of childhood sexual abuse. Journal of Affective Disorders 2021; 282:272–9. Available from: URL: <https://www.sciencedirect.com/science/article/pii/s0165032720332717>.

Liu Y, Li M, Gao Y, Zhang C, Wang Y, Liu X, Yang S, Li J. Specific correlation between childhood trauma and social cognition in Chinese Han first-episode, drug-naive major depressive disorder. Journal of Affective Disorders 2023; 333:51-57.

Minnich AM, Gordon KH, Kwan MY, Troop-Gordon W. Examining the mediating role of alexithymia in the association between childhood neglect and disordered eating behaviors in men and women. Psychology of Men & Masculinity 2017; 18(4):414–21.

Mitchell KS, Mazzeo SE. Mediators of the association between abuse and disordered eating in undergraduate men. Eating Behaviors 2005; 6(4):318–27. Available from: URL: <https://www.sciencedirect.com/science/article/pii/s1471015305000140>.

Mlotek AE. Contributions of Emotional Competence to the Link between Childhood Maltreatment and Adult Attachment; 2019. Available from: URL: https://search.proquest.com/openview/9f534d2baba044462103409c8dc05191/1?pq-origsite=gscholar&cbl=18750&diss=y.

Morie KP, Zhai ZW, Potenza MN, Mayes LC. Alexithymia, Emotion-Regulation Strategies, and Traumatic Experiences in Prenatally Cocaine-Exposed Young Adults. The American Journal on Addictions 2020; 29(6):492–9.

Paivio SC, McCulloch CR. Alexithymia as a mediator between childhood trauma and self-injurious behaviors. Child Abuse & Neglect 2004; 28(3):339–54. Available from: URL: https://www.sciencedirect.com/science/article/pii/S0145213404000341.

Senkal I, Isikli S. Childhood Traumas and Attachment Style - Associated Depression Symptoms: The Mediator Role of Alexithymia. Turkish Journal of Psychiatry 2015.

Spitzer C, Busche W, Vogel M, Barnow S, Freyberger H, Grabe HJ. Elterliches Erziehungsverhalten, Kindesmisshandlung und Alexithymie. pdp 2009; 8(1):13–22. Available from: URL: https://elibrary.klett-cotta.de/article/99.120110/pdp-8-1-13.

Strodl E, Wylie L. Childhood trauma and disordered eating: Exploring the role of alexithymia and beliefs about emotions. Appetite 2020; 154:104802. Available from: URL: https://www.sciencedirect.com/science/article/pii/s0195666320303391.

Terock J, van der Auwera S, Janowitz D, Spitzer C, Barnow S, Miertsch M et al. From Childhood Trauma to Adult Dissociation: The Role of PTSD and Alexithymia. Psychopathology 2016; 49(5):374–82.

Terock J, van der Auwera S, Janowitz D, Wittfeld K, Frenzel S, Klinger-König J et al. Childhood trauma and adult declarative memory performance in the general population: The mediating effect of alexithymia. Child Abuse & Neglect 2020; 101:104311. Available from: URL: https://www.sciencedirect.com/science/article/pii/s0145213419304879.

Xie Z-M, Fang Y, Mai Y-L, Zhao J-B, Zhang X-Y, Zhao J-B. The role of alexithymia in childhood trauma and suicide risk: A multi-group comparison between left-behind experience students and no left-behind experience students. Personality and Individual Differences 2021; 172:110260. Available from: URL: https://www.sciencedirect.com/science/article/pii/s0191886920304499.

Zhang CH, Li G, Fan ZY, Tang XJ, Zhang F. Psychological capital mediating the relationship between childhood trauma and alexithymia in Chinese medical students: a cross-sectional study. Psychology Research and Behavior Management 2020:1343–52.

Zhang H, Yang C, Ou Y, Tan Y, Wu S, Hu M et al. Relationship between childhood trauma and suicidal risk in prisoners: the mediating effect of alexithymia. Journal of Southern Medical University 2021; (41(2)):299–304. Available from: URL: https://europepmc.org/article/med/33624606.

Zou Z, Huang Y, Wang J, He Y, Min W, Chen X et al. Association of childhood trauma and panic symptom severity in panic disorder: Exploring the mediating role of alexithymia. Journal of Affective Disorders 2016; 206:133–9. Available from: URL: <https://www.sciencedirect.com/science/article/pii/s0165032715311794>.

## Articles included in the meta-analysis between TAS and SCL/BSI

Barbosa F, Freitas J, Barbosa A. Alexithymia in chronic urticaria patients. Psychology, Health & Medicine 2011; 16(2):215–24.

Bilge Y, Bilge Y, Emiral E, Özkars BN. Examination of the relationships among psychological symptoms, alexithymia and emotional regulation: mediating role of emotion regulation. 1300-8773 2018; (0):1. Available from: URL: http://openaccess.izu.edu.tr/xmlui/handle/20.500.12436/850.

Bilotta E, Giacomantonio M, Leone L, Mancini F, Coriale G. Being alexithymic: Necessity or convenience. Negative emotionality × avoidant coping interactions and alexithymia. Psychology and

Conrad R, Weber NF, Lehnert M, Holz FG, Liedtke R, Eter N. Alexithymia and emotional distress in patients with central serous chorioretinopathy. Psychosomatics 2007; 48(6):489–95. Available from: URL: https://www.sciencedirect.com/science/article/pii/s0033318207709854.

Evren C, Dalbudak E, Çakmak D. Alexithymia and Personality in Relation to Dimensions of Psychopathology in Male Alcohol-Dependent Inpatients; 2008. Available from: URL: https://www.psychiatry-psychopharmacology.com/content/files/sayilar/63/18_1_1.pdf.

Flasbeck V, Popkirov S, Brüne M. Frontal EEG asymmetry in borderline personality disorder is associated with alexithymia. bord personal disord emot dysregul 2017; 4(1):20. Available from: URL: https://bpded.biomedcentral.com/articles/10.1186/s40479-017-0071-7.

Imperatori C, Della Marca G, Brunetti R, Carbone GA, Massullo C, Valenti EM et al. Default Mode Network alterations in alexithymia: an EEG power spectra and connectivity study. Sci Rep 2016; 6(1):36653. Available from: URL: https://www.nature.com/articles/srep36653.

Jones MP, Schettler A, Olden K, Crowell MD. Alexithymia and somatosensory amplification in functional dyspepsia. Psychosomatics 2004; 45(6):508–16. Available from: URL: https://www.sciencedirect.com/science/article/pii/s0033318204701425.

Karaca Dinç P, Oktay S, Durak Batıgün A. Mediation role of alexithymia, sensory processing sensitivity and emotional-mental processes between childhood trauma and adult psychopathology: a self-report study. BMC Psychiatry 2021; 21(1):508. Available from: URL: <https://link.springer.com/article/10.1186/s12888-021-03532-4>.

Kahramanol B, Dag I. Alexithymia, anger and anger expression styles as predictors of psychological symptoms. Dusunen Adam 2018; 31(1):30–9.

Kerr S, Johnson VK, Gans SE, Krumrine J. Predicting Adjustment During the Transition to College: lexithymia, Perceived Stress, and Psychological Symptoms. Journal of College Student Development 004; 45(6):593–611. Available from: URL: https://muse.jhu.edu/pub/1/article/175649/summary.

Korkoliakou P, Efstathiou V, Giannopoulou I, Christodoulou C, Kouris A, Rigopoulos D et al. Psychopathology and alexithymia in patients with psoriasis. An. Bras. Dermatol. 2017; 92(4):510–5.

Köse O, Sayar K, Ebrinc S. Psychometric assessment of alopecia areata patients before and after dermatological treatment. n Bulletin of Clinical Psychopharmacology 2000. Available from: URL: <https://psychiatry-psychopharmacology.com/content/files/sayilar/102/10_1_4.pdf>.

Leweke F, Bausch S, Leichsenring F, Walter B, Stingl M. Alexithymia as a predictor of outcome of psychodynamically oriented inpatient treatment. Psychother Res 2009; 19(3):323–31.

Li X, He L, Liu J, Guo W, Wang Q, Fang P et al. The rs6311 of serotonin receptor 2A (5-HT2A) gene is associated with alexithymia and mental health. Journal of Affective Disorders 2020; 272:277–82. Available from: URL: <https://www.sciencedirect.com/science/article/pii/s0165032719335360>.

Li R, Kajanoja J, Lindblom J, Korja R, Karlsson L, Karlsson H, Nolvi S, Karukivi M. The role of alexithymia and perceived stress in mental health responses to COVID-19: a conditional process model. Journal of Affective Disorders 2022; 306, 9-18.

Liang B, West J. Relational health, alexithymia, and psychological distress in college women: testing a mediator model. American Journal of Orthopsychiatry 2011; 81(2):246–54.

Linn BK, Zhao J, Bradizza CM, Lucke JF, Ruszczyk MU, Stasiewicz PR. Alexithymia disrupts emotion regulation processes and is associated with greater negative affect and alcohol problems. Journal of Clinical Psychology 2021; 77(12):2915–28.

Ludwig G, Krenz S, Zdrojewski C, Bot M, Rousselle I, Stagno D et al. Psychodynamic interventions in cancer care I: psychometric results of a randomized controlled trial 2013.

Mannarini S, Kleinbub JR. Parental-Bonding and Alexithymia in Adolescents with Anorexia Nervosa, Their Parents, and Siblings. Behavioral Sciences 2022; 12(5):123. Available from: URL: https://www.mdpi.com/1601592.

Martinez-Sanchez F, Fernandez-Castro J, Aparicio MC. Comparison of alexithymia and personal competence as moderators of stress reactions between students and teachers. Studia Psychologica 2004; 46:1.

Mikolajczak M, Luminet O. Is alexithymia affected by situational stress or is it a stable trait related to emotion regulation? Personality and Individual Differences 2006; 40(7):1399–408. Available from: URL: https://www.sciencedirect.com/science/article/pii/s0191886905004083.

Panfilis C de, Ossola P, Tonna M, Catania L, Marchesi C. Finding words for feelings: The relationship between personality disorders and alexithymia. Personality and Individual Differences 2015; 74:285–91. Available from: URL: https://www.sciencedirect.com/science/article/pii/s0191886914006308.

Pedrosa Gil F, Bidlingmaier M, Ridout N, Scheidt CE, Caton S, Schoechlin C et al. The relationship between alexithymia and salivary cortisol levels in somatoform disorders. Nordic Journal of Psychiatry 2008; 62(5):366–73.

Pedrosa Gil F, Weigl M, Wessels T, Irnich D, Baumüller E, Winkelmann A. Parental bonding and alexithymia in adults with fibromyalgia. Psychosomatics 2008; 49(2):115–22. Available from: URL: https://www.sciencedirect.com/science/article/pii/s0033318208709496.

Porcelli P, Affatati V, Bellomo A, Carne M de, Todarello O, Taylor GJ. Alexithymia and psychopathology in patients with psychiatric and functional gastrointestinal disorders. Psychotherapy and Psychosomatics 2004; 73(2):84–91.

Renzi A, Solano L, Di Trani M, Ginobbi F, Minutolo E, Tambelli R. The effects of an expressive writing intervention on pregnancy rates, alexithymia and psychophysical health during an assisted reproductive treatment. Psychology & Health 2020; 35(6):718–33.

Ritzl A, Csukly G, Balázs K, Égerházi A. Facial emotion recognition deficits and alexithymia in borderline, narcissistic, and histrionic personality disorders. Psychiatry Research 2018; 270:154–9. Available from: URL: https://www.sciencedirect.com/science/article/pii/s0165178117320139.

Saarijärvi S, Salminen JK, Toikka TB. Alexithymia and depression: a 1-year follow-up study in outpatients with major depression. Journal of psychosomatic research 2001; 51(6):729–33. Available from: URL: https://www.sciencedirect.com/science/article/pii/s0022399901002574.

Salcuni S, Mancinelli E, Muneratti A, Grillo A, Alessi C, Guglielmino A et al. Couples undergoing Assisted Reproductive Techniques: An Actor-Partner Interdependence Model of dyadic adjustment, attachment, and body-image avoidance. Health Psychology Open 2021; 8(2):20551029211039923.

Sayar K, Köse O, Ebrinç S, Şetin M. Hopelessness, Depression and Alexithymia in Young Turkish Soldiers Suffering from Alopecia areata. Dermatology and Psychosomatics / Dermatologie und Psychosomatik 2001; 2(1):12–5.

Schäfer R, Schneider C, Sitte W, Franz M. Validitätshinweise der Deutschen Version der TAS-20 - Beitrag zur 52. Arbeitstagung des Deutschen Kollegiums für Psychosomatische Medizin in Bad Honnef (2001). Psychotherapie, Psychosomatik, medizinische Psychologie 2002; 52(11):449–53. Available from: URL: https://www.thieme-connect.com/products/ejournals/html/10.1055/s-2002-35279.

Simonsen S, Eikenaes IU-M, Bach B, Kvarstein E, Gondan M, Møller SB et al. Level of alexithymia as a measure of personality dysfunction in avoidant personality disorder. Nordic Journal of Psychiatry 2021; 75(4):266–74.

Simson U, Martin K, Schäfer R, Janssen P, Franz M. Alexithymie in einer klinischen Stichprobe. Psychotherapie, Psychosomatik, medizinische Psychologie 2005; 55(7):347–53. Available from: URL: https://www.thieme-connect.com/products/ejournals/html/10.1055/s-2004-834751.

Simson U, Martin K, Schäfer R, Franz M, Janssen P. Veränderung der Wahrnehmung von Emotionen im Verlauf stationärer psychotherapeutischer Behandlung. Psychotherapie, Psychosomatik, medizinische Psychologie 2006; 56(9-10):376–82. Available from: URL: <https://www.thieme-connect.com/products/ejournals/html/10.1055/s-2006-940127>.

Subic-Wrana C, Bruder S, Thomas W, Gaus E, Merkle W, Köhle K. Verteilung des Persönlichkeitsmerkmals Alexithymie bei Patienten in stationärer psychosomatischer Behandlung - gemessen mit TAS-20 und LEAS. Psychotherapie, Psychosomatik, medizinische Psychologie 2002; 52(11):454–60. Available from: URL: https://www.thieme-connect.com/products/ejournals/html/10.1055/s-2002-35281.

Subic-Wrana C, Bruder S, Thomas W, Lane RD, Köhle K. Emotional awareness deficits in inpatients of a psychosomatic ward: a comparison of two different measures of alexithymia. Psychosomatic Medicine 2005; 67(3):483–9. Available from: URL: https://journals.lww.com/psychosomaticmedicine/fulltext/2005/05000/emotional_awareness_deficits_in_inpatients_of_a.21.aspx.

Tran US, Glück TM, Nader IW. Investigating the Five Facet Mindfulness Questionnaire (FFMQ): construction of a short form and evidence of a two-factor higher order structure of mindfulness. Journal of Clinical Psychology 2013; 69(9):951–65.

Tran US, Walter T, Remmel A. Faktoren psychosozialer Beeinträchtigung. Diagnostica 2012; 58(2):75–86.

Viganò CA, Beltrami MM, Bosi MF, Zanello R, Valtorta M, Maconi G. Alexithymia and Psychopathology in Patients Suffering From Inflammatory Bowel Disease: Arising Differences and Correlations to Tailoring Therapeutic Strategies. Front. Psychiatry 2018; 9:324.

Vittori A, Marinangeli F, Bignami EG, Simonini A, Vergallo A, Fiore G et al. Analysis on Burnout, Job Conditions, Alexithymia, and Other Psychological Symptoms in a Sample of Italian Anesthesiologists and Intensivists, Assessed Just before the COVID-19 Pandemic: An AAROI-EMAC Study. Healthcare 2022; 10(8):1370. Available from: URL: https://www.mdpi.com/2227-9032/10/8/1370.

Wingbermühle E, Egger JIM, Verhoeven WMA, van der Burgt I, Kessels RPC. Affective functioning and social cognition in Noonan syndrome. Psychol. Med. 2012; 42(2):419–26.

Zeeck A, Stelzer N, Linster HW, Joos A, Hartmann A. Emotion and eating in binge eating disorder and obesity. European Eating Disorders Review 2011; 19(5):426–37.

# Supplementary Figures and Tables

## Supplementary Figures

**Figure 1**

Origin of participants based on origin country of the studies (CTQ-TAS)

**Figure 2**

Origin of participants based on origin country of the studies (TAS-SCL)

*Note.* Turkey was listed as Asian country.

**Figure 3**

Forest Plot (Emotional Neglect)


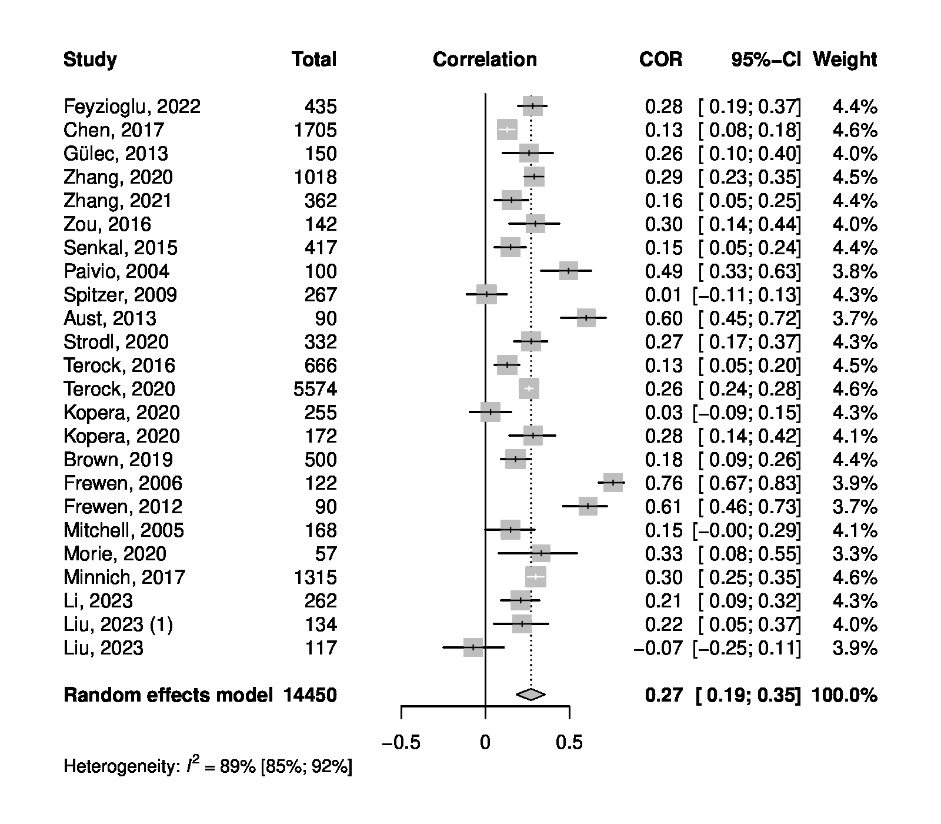


**Figure 4**

Funnel Plot (Emotional Neglect)


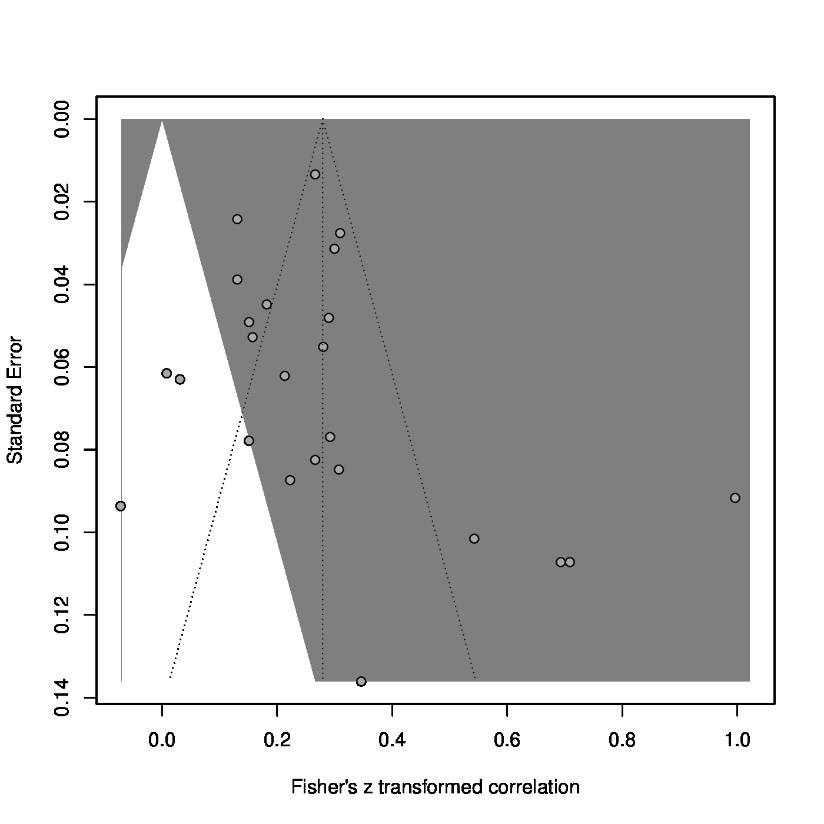


**Figure 5**

Forest Plot (Emotional Abuse)


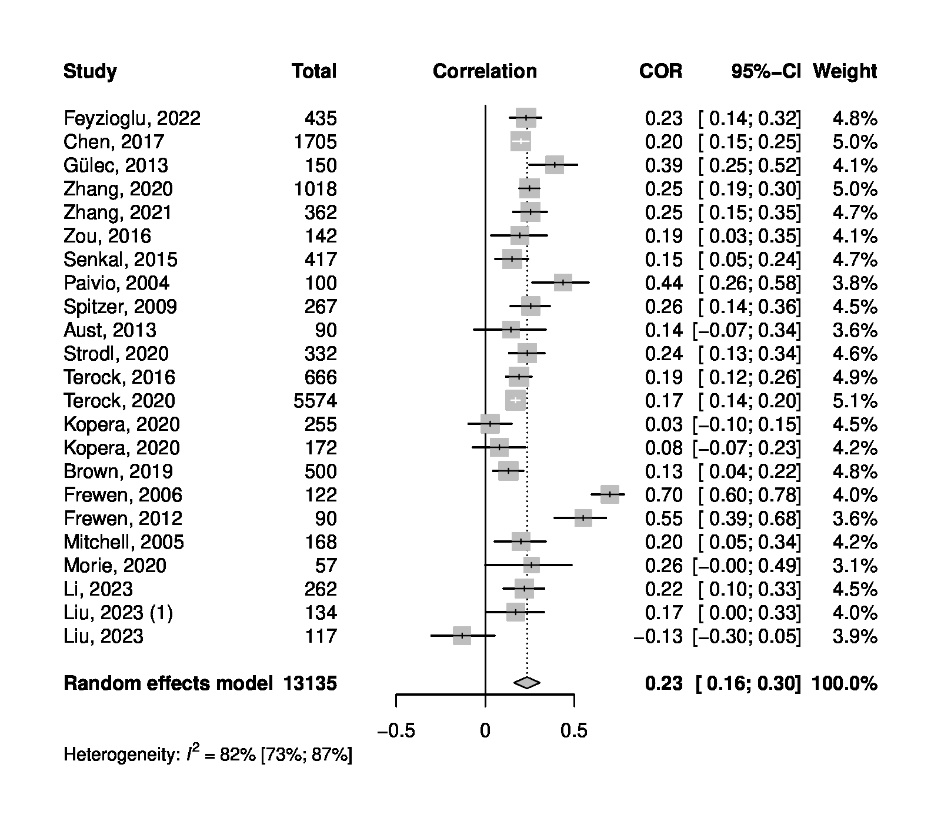


**Figure 6**

Funnel Plot (Emotional Abuse)


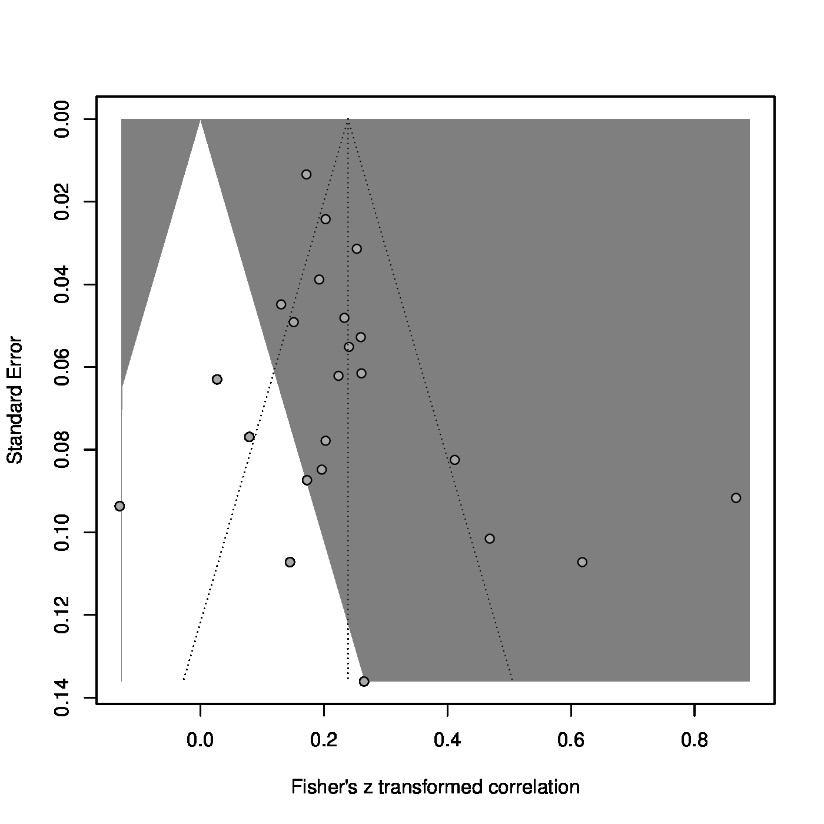


**Figure 7**

Forest Plot (Sexual Abuse)


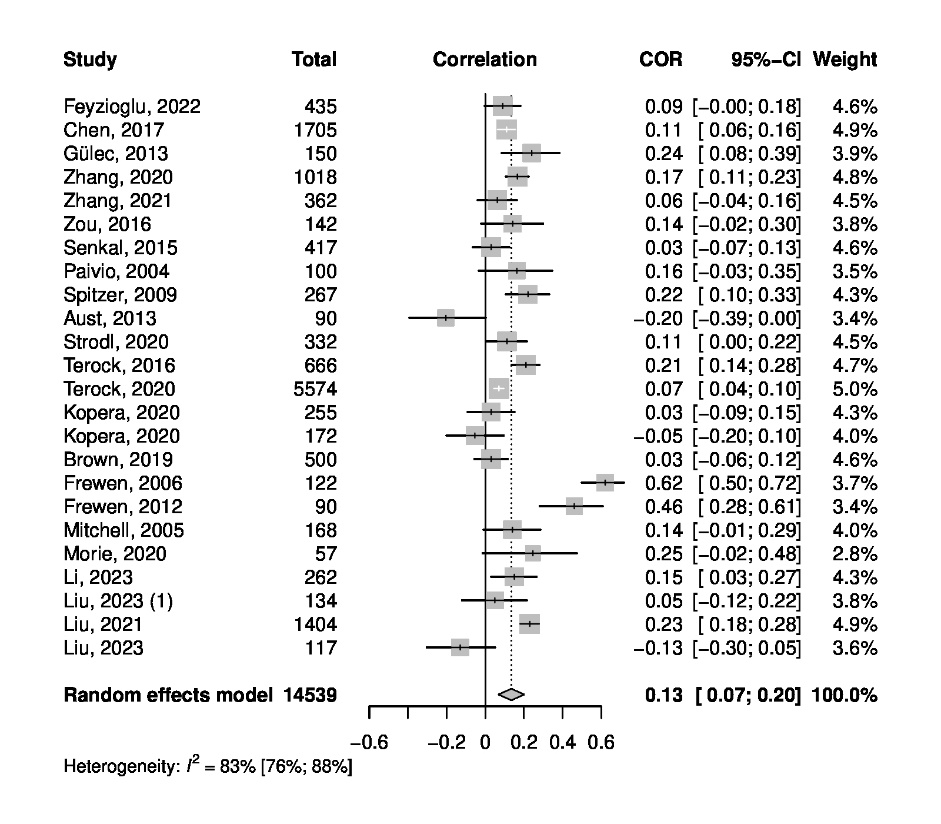


**Figure 8**

Funnel Plot (Sexual Abuse)


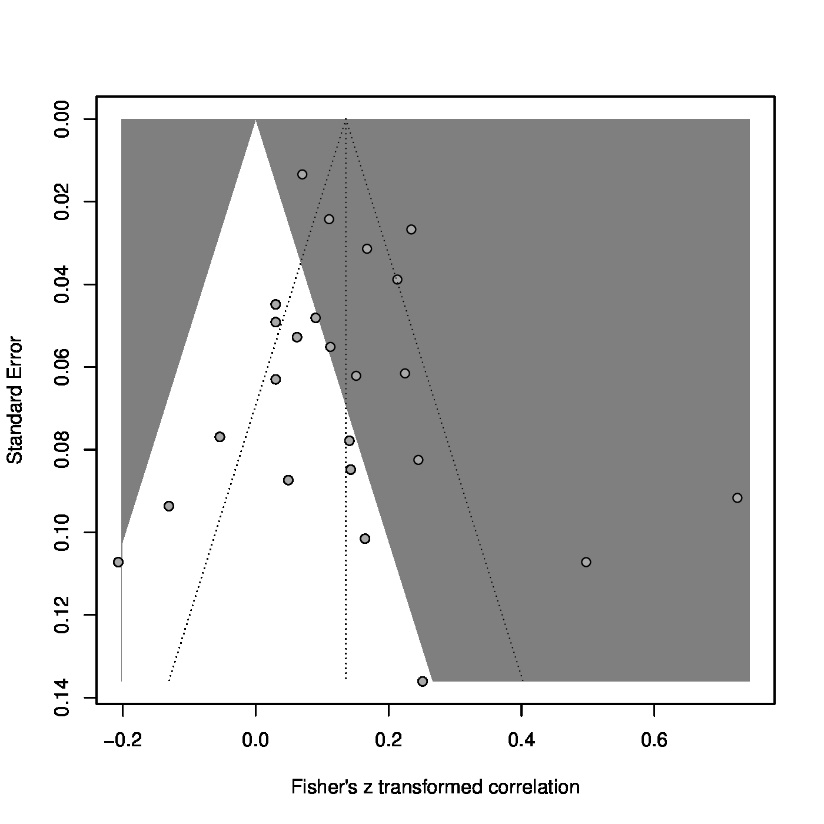


**Figure 9**

Forest Plot (Physical Abuse)


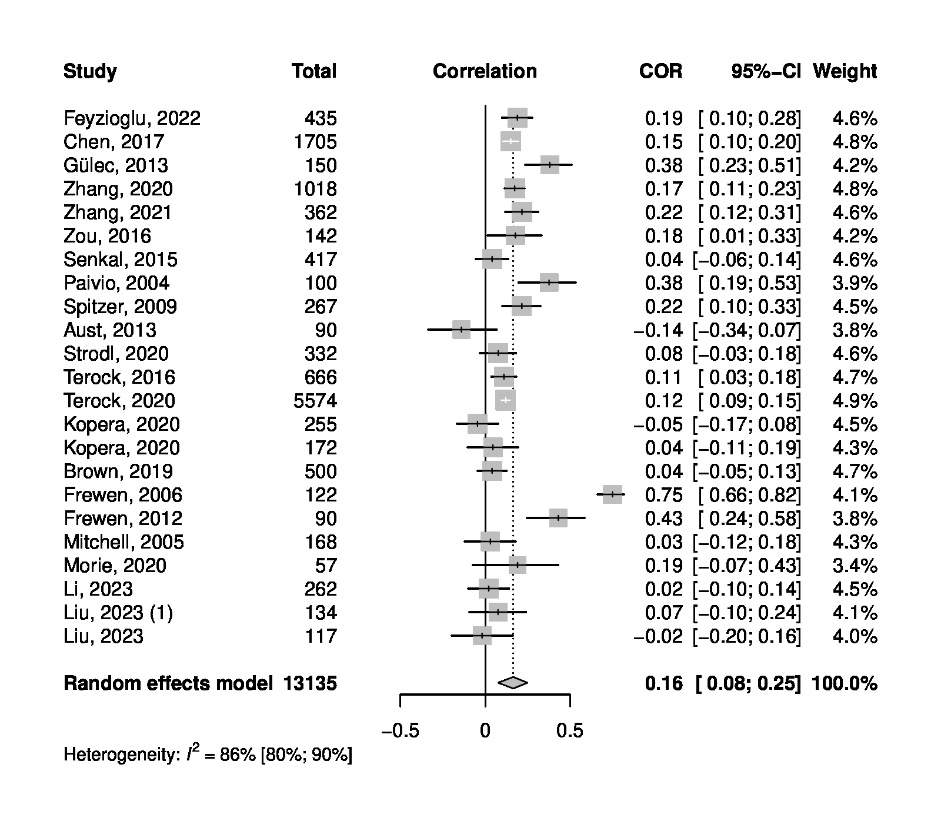


**Figure 10**

Funnel Plot (Physical Abuse)


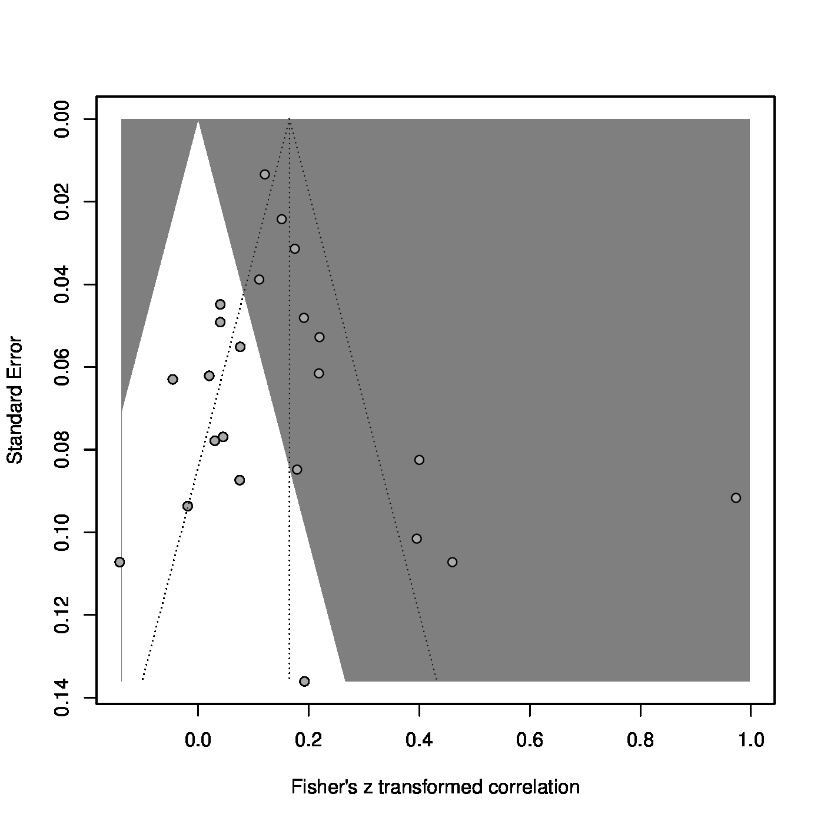


**Figure 11**

Forest Plot (Physical Neglect)


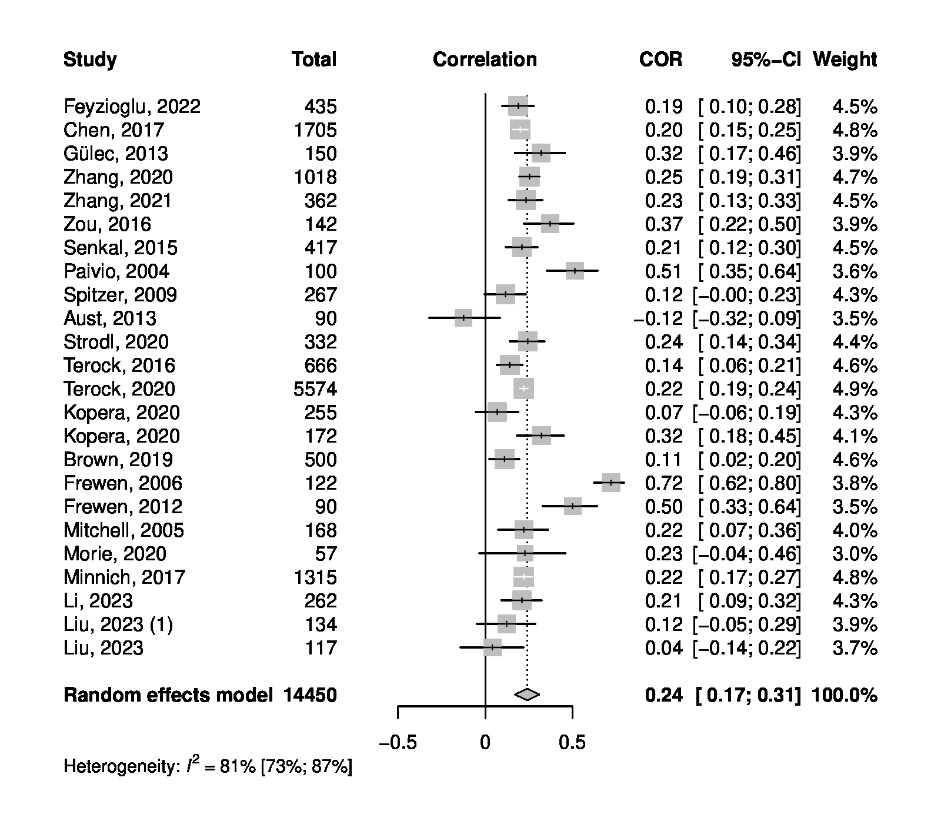


**Figure 12**

Funnel Plot (Physical Neglect)


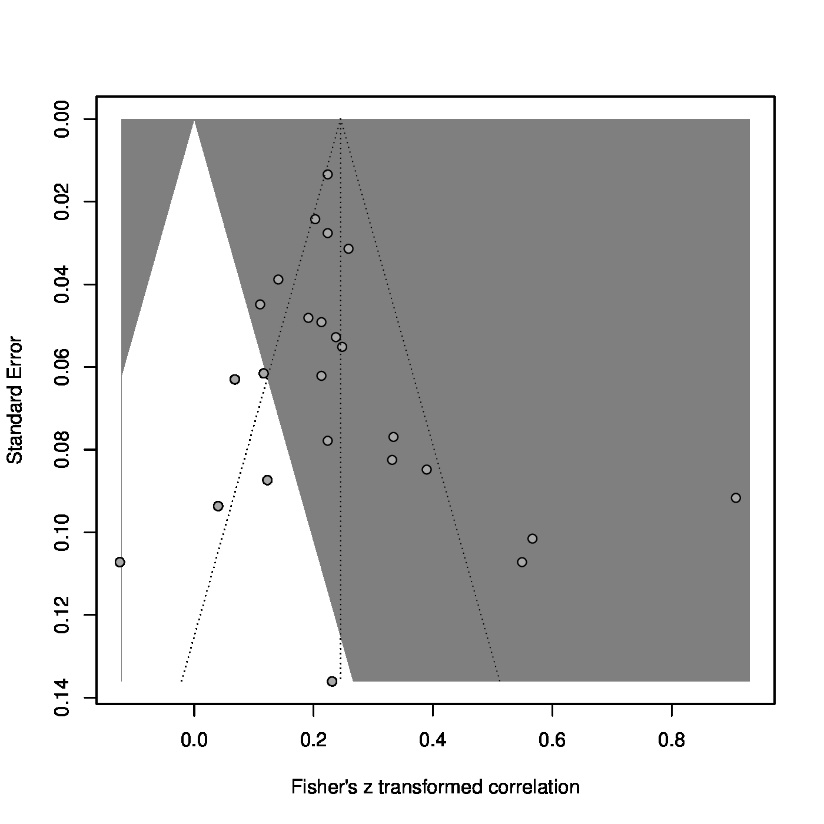


## Supplementary Tables

**Table 1**

Keywords for article search (child abuse and alexithymia)

| **Database** | **Number of articles** | **Keywords** |
| --- | --- | --- |
| PubMed | 143 | "alexithymia" AND ((child abuse) OR (child neglect) OR (child maltreatment)) |
| PsycINFO  (via EBSCOhost) | 167 | Alexithymia AND child abuse or child neglect or child maltreatment |
| PSYNDEX  (via EBSCOhost) | 11 | Alexithymia AND child abuse or child neglect or child maltreatment |
| Google Scholar | 7570 – only the first 200 screened | "alexithymia" AND ("child abuse" OR "child neglect" OR "child maltreatment") |

**Table 2**

Keywords for article search (alexithymia and psychopathology)

| **Database** | **Number of articles** | **Keywords** |
| --- | --- | --- |
| PubMed | 91 | ("alexithymia") AND ((BSI) OR (SCL-90) OR (SCL-90-R)) |
| PsycINFO  (via EBSCOhost) | 112 | Alexithymia AND SCL-90 or SCL-90-r or BSI |
| PSYNDEX  (via EBSCOhost) | 57 | Alexithymia AND SCL-90 or SCL-90-r or BSI |
| Google Scholar | 4710 – only the first 200 screened | "alexithymia" AND ("SCL-90" OR "SCL-90-R" OR "BSI") |

# Subgroup Analyses and Meta-Regressions

## CTQ-TAS

**Table 3**

Subgroup analysis for healthy samples vs. samples with mental disorders

| **Sample Characteristics** | ***k*** | ***r*** | **95%-CI** | **I^2^** |
| --- | --- | --- | --- | --- |
| Healthy | 15 | .30 | [.25; .35] | 83.9 % |
| Mental Disorder | 8 | .15 | [.04; .26] | 73.2 % |

*Note.* Unclear samples were removed from the analysis. Test for subgroup differences (random effects model) revealed a significant difference (*Q* = 6.13, *df* = 1, *p* = .01).

**Table 4**

Model results for a meta-regression with mean age as predictor for the correlation between CTQ and TAS

|  | ***β*** | ***SE*** | ***p*** |
| --- | --- | --- | --- |
| Intercept | .42 | .10 | <.01* |
| Mean Age | < -.01 | < .01 | .12 |

*Note. k* = 26; * indicates significance with α = .05.

**Table 5**

Model results for a meta-regression with mean TAS score as predictor for the correlation between CTQ and TAS

|  | ***β*** | ***SE*** | ***p*** |
| --- | --- | --- | --- |
| Intercept | .55 | .20 | .01* |
| Mean TAS score | -.01 | < .01 | .14 |

*Note. k* = 21; * indicates significance with α = .05.

**Table 6**

Model results for a meta-regression with percentage of female participants as predictor for the correlation between CTQ and TAS

|  | ***β*** | ***SE*** | ***p*** |
| --- | --- | --- | --- |
| Intercept | .18 | .06 | <.01* |
| % female | < .01 | < .01 | .13 |

*Note. k* = 28; * indicates significance with α = .05.

## TAS-SCL/BSI

**Table 7**

Subgroup analysis for healthy samples vs. samples with mental disorders vs. samples with physical disorders

| **Sample Characteristics** | ***k*** | ***r*** | **95%-CI** | **I^2^** |
| --- | --- | --- | --- | --- |
| Healthy | 21 | .44 | [.39; .49] | 79.8% |
| Mental Disorder | 15 | .44 | [.39; .50] | 62.6% |
| Physical Disorder | 8 | .30 | [.04; .53] | 92.1% |

*Note.* Unclear samples were removed from the analysis. Test for subgroup differences (random effects model) revealed no significant differences (*Q* = 1.32, *df* = 2, *p* = .52).

**Table 8**

Model results for a meta-regression with mean age as predictor for the correlation between TAS and SCL/BSI

|  | ***β*** | ***SE*** | ***p*** |
| --- | --- | --- | --- |
| Intercept | .45 | .10 | <.0001* |
| Mean Age | < .001 | < .01 | .71 |

*Note. k* = 39; * indicates significance with α = .05.

**Table 9**

Model results for a meta-regression with mean TAS score as predictor for the correlation between TAS and SCL/BSI

|  | ***β*** | ***SE*** | ***p*** |
| --- | --- | --- | --- |
| Intercept | .22 | .19 | .28 |
| Mean TAS score | < .01 | < .001 | .21 |

*Note. k* = 28.

**Table 10**

Model results for a meta-regression with percentage of female participants as predictor for the correlation between TAS and SCL/BSI

|  | ***β*** | ***SE*** | ***p*** |
| --- | --- | --- | --- |
| Intercept | .51 | .08 | <.0001* |
| % female | < -.001 | < .01 | .89 |

*Note. k* = 44; * indicates significance with α = .05.
